# Energy-Aware adaptive virtualization and migration protocol for green IoT wireless sensor networks

**DOI:** 10.1038/s41598-025-28783-z

**Published:** 2025-12-29

**Authors:** Yi liu, Yan Li, Nianming Ge

**Affiliations:** School of Electronic Information Engineering, Sanjiang University, Nanjing, 210000 Jiangsu Province China

**Keywords:** Green IoT, Wireless sensor networks (WSN), Energy-Aware, Virtualization, Resource migration, Federal deep reinforcement learning (FDRL), Energy science and technology, Engineering, Mathematics and computing

## Abstract

The swift expansion of the Internet of Things (IoT) has resulted in heightened energy requirements and sustainability issues inside extensive wireless sensor networks. This research presents the Energy-Aware Adaptive Virtualization and Migration (EAVM) protocol to tackle these difficulties in Green IoT-based Wireless Sensor Networks. The technique combines Federated Deep Reinforcement Learning (FDRL) with hybrid solar–RF energy harvesting to facilitate intelligent and sustainable resource management. EAVM allocates and migrates virtual resources dynamically according to real-time energy conditions, ensuring workload balance and extended network stability. A thorough simulation methodology assesses its performance relative to contemporary state-of-the-art techniques, illustrating that EAVM attains enhanced energy efficiency, scalability, and sustainability within dynamic IoT systems.

## Introduction

The Internet of Things has revolutionized connectivity by enabling seamless data exchange across various devices, supporting applications from smart cities to environmental monitoring. Green IoT, an emerging concept, integrates sustainability principles into IoT ecosystems, particularly inside WSNs, which are crucial for data collection in resource-constrained environments^1^. WSNs, composed of multiple sensor nodes, face significant energy limitations due to their reliance on battery power, leading to rapid depletion and reduced network longevity^2^. Virtualization methods abstract physical resources into virtual entities, enabling dynamic allocation to improve performance^3^. Resource migration enhances efficiency by reallocating virtual resources between nodes to balance computational workloads^4^. In Green IoT, these techniques are adapted to minimize energy consumption and environmental effects, aligning with global sustainability goals^5^. Recent advancements, such as FDRL, provide intelligent resource management by simulating complex network dynamics in a decentralized manner^6,7^. Energy harvesting systems, which capture ambient energy from solar, thermal, or kinetic sources, provide sustainable power options, extending node lifespans and reducing ecological consequences^8^.

The significance of energy-aware virtualization and migration in Green IoT lies in its ability to enhance system efficiency while minimizing environmental impacts. With IoT devices projected to reach billions by 2030, efficient resource management is essential to sustain service quality while minimizing energy consumption^9^. In practical applications, Green IoT has been extensively utilized in smart homes for intelligent lighting and climate control systems that minimize energy consumption, in smart transportation for optimizing fuel efficiency and traffic management, in healthcare for ongoing patient monitoring with energy-efficient sensors, and in precision agriculture for sustainable irrigation and soil management^10–12^.

In smart grid applications, these techniques facilitate real-time monitoring with little power consumption, hence allowing for the integration of renewable energy^13^. In precision agriculture, energy-efficient wireless sensor networks enable prolonged sensor deployment, optimizing resource utilization and promoting sustainable agricultural methods^14^. These solutions economically reduce operational costs in sectors like logistics by extending the lifespan of equipment^15^. They enable IoT deployment in energy-scarce regions, fostering equitable access to technology while adhering to environmental rules^16^.

Several challenges persist in the implementation of adaptive virtualization and migration in Green IoT Wireless Sensor Networks. The energy consumption linked to migration activities, which entail the transfer of virtual computers or containers, can significantly diminish battery resources^17^. Scalability difficulties arise in dense installations, when an increased number of devices leads to network congestion and delayed resource allocation^18^. Security weaknesses in FDRL-based systems create avenues for attacks, endangering data integrity^19^. Energy harvesting is impeded by intermittency and low conversion efficiency, limiting its reliability in dynamic environments^20^. The variety of IoT devices influences the standardization of virtualization frameworks^21^. Moreover, traditional centralized resource management often demonstrates inefficacy in distributed wireless sensor networks, leading to suboptimal energy utilization^22^. These challenges necessitate innovative, energy-efficient methods that thoroughly address technical and environmental constraints^23^.

Recent studies in green cloud computing have investigated the optimization of service migration. A novel strategy to diminish energy consumption in the prioritized live migration of services, grounded in green cloud computing, has shown that emphasizing service significance during migration can substantially reduce energy overhead while ensuring service continuity, in accordance with the energy-aware principles of the proposed EAVM framework^24^.

This research is driven by the urgent need to reconcile escalating IoT service demands with sustainability imperatives. Contemporary techniques often overlook the incorporation of virtualization, migration, and energy harvesting, resulting in inefficient resource utilization and negative environmental impacts^17^. The proliferation of energy-intensive IoT applications and the ecological consequences of traditional network operations drive the pursuit of an integrated framework employing advanced learning and renewable energy technologies.

The primary objective of this study is to present an EAVM protocol for Green IoT Wireless Sensor Networks. This protocol aims to improve the allocation and migration of virtual resources through sophisticated, dynamic algorithms that emphasize energy efficiency and sustainability. The breakthrough involves integrating FDRL with energy harvesting technology, enabling proactive decision-making that adapts to real-time network conditions. EAVM use FDRL to model interactions among user connectivity, resource demands, and energy availability, facilitating predictive migrations that minimize latency and power consumption. The protocol incorporates ambient energy sources to facilitate self-sustaining operations, hence reducing battery reliance and the network’s carbon footprint.

The proposed EAVM protocol differs from current FDRL-based optimization frameworks, which primarily emphasize resource allocation or communication efficiency, by offering a cohesive integration of virtualization, migration, and hybrid energy harvesting inside a singular adaptive framework. This integration enables EAVM to execute energy-conscious and anticipatory resource movement among nodes, attaining both operational efficacy and ecological sustainability, a topic previously unexamined in existing literature.

The main contributions of this study are summarized as follows:

- **A unified energy-aware framework (EAVM)**: The research presents an innovative protocol that concurrently combines virtualization, migration, and hybrid solar–RF energy harvesting in Green IoT-based Wireless Sensor Networks (WSNs).
- **Intelligent resource management via FDRL**: The suggested method utilizes Federated Deep Reinforcement Learning to facilitate distributed and adaptive decision-making for the optimization of dynamic energy and workload.
- **Cross-layer coordination mechanism**: EAVM integrates energy harvesting, job relocation, and resource allocation via a multi-layer adaptive control technique, thereby improving sustainability and performance stability.
- **Scalable and self-optimizing architecture**: The protocol is engineered to facilitate extensive IoT implementations with reduced communication overhead and elevated fault tolerance amid variable energy conditions.
- **Comprehensive validation**: A comprehensive simulation framework validates the protocol’s capacity to sustain high efficiency, optimized energy consumption, and extended network longevity in comparison to current leading methodologies.

The article is structured as follows. Section 2 analyzes relevant literature, emphasizing the existing deficiencies in the research. Section 3 outlines the EAVM system paradigm and its foundational assumptions. Section 4 outlines the proposed protocol, including algorithmic details. Section 5 evaluates performance using simulations, contrasting EAVM with state-of-the-art techniques. Section 6 concludes the analysis and outlines potential research directions.

## Related works

Recent developments in Green IoT and WSNs have emphasized energy-efficient resource management to improve sustainability. Virtualization approaches have proved essential in transforming real resources into controllable virtual entities, facilitating efficient allocation in resource-limited IoT scenarios. A lightweight virtualization framework for WSNs was suggested, partitioning sensor nodes into virtual instances to enhance job execution, resulting in a 10% reduction in energy consumption via resource isolation^25^. This technique, however, lacked dynamic migration, constraining its adaptability to fluctuating network conditions. A recent study proposed a migration technique for IoT edge devices that employs predictive analytics to reduce latency during resource relocation, achieving a 15% enhancement in energy efficiency in dense deployments^26^. Notwithstanding these developments, centralized decision-making in such protocols frequently results in scalability constraints, especially in extensive Green IoT networks necessitating distributed control^27^.

The incorporation of machine learning, specifically Federated Learning (FL) and Deep Reinforcement Learning (DRL), has revolutionized resource management in sustainable IoT systems. A 2023 study utilized federated learning to enhance energy-efficient task allocation in wireless sensor networks, facilitating decentralized model training while maintaining privacy^28^. Furthermore, DRL has been utilized to tackle dynamic IoT situations. A DRL-based virtual machine placement technique dynamically changed resource allocations in response to workload variations^29^. FDRL, which integrates FL and DRL, has demonstrated potential for collaborative decision-making in heterogeneous WSNs. A recent study employed FDRL to enhance traffic offloading in IoT networks, achieving a 12% reduction in latency while optimizing energy distribution compared to conventional RL methods^30^. Nonetheless, these studies frequently emphasize communication efficiency at the expense of sustainability indicators, such as carbon footprint reduction, and seldom integrate energy harvesting into their frameworks^31^.

Energy harvesting technologies have emerged as fundamental for sustainable wireless sensor networks, utilizing ambient energy to prolong node lifespans and reduce environmental effect. A 2024 study introduced a hybrid harvesting technique that combines solar and RF energy sources, focusing on enhancing the operational lifespan of outdoor IoT sensors^32^. Another innovation entailed the utilization of predictive algorithms to enhance energy harvesting schedules in accordance with environmental circumstances^33^. These strategies facilitate energy-conscious resource allocation when integrated with virtualization. A 2023 protocol adjusted virtual resources in harvested wireless sensor networks based on available energy, appropriate for agricultural Internet of Things applications^34^. Migration within harvested systems increases efficiency by transferring jobs to nodes with abundant energy. A recent methodology integrated harvesting rates into migration strategies, resulting in balanced loads and a 15% decrease in outages^35^. Nonetheless, these approaches frequently regard virtualization and migration as distinct processes, lacking an integrated framework that utilizes FDRL for adaptive and sustainable management^36^.

Despite significant gains, the existing research reveals certain limits. Many studies highlight performance metrics such as latency and throughput, sometimes neglecting sustainability criteria vital for Green IoT^26,29^. Centralized designs continue to limit scalability in decentralized wireless sensor networks, where node autonomy is essential^28^. FDRL solutions, despite their potential, rarely address the integration of virtualization and migration in energy-harvested systems, leading to shortcomings in holistic resource management^30^. Security concerns, particularly data exposure during migrations in federated systems, are inadequately addressed^31^. The irregularity of energy harvesting obstructs effective resource distribution, as current models do not respond to unexpected energy fluctuations. These deficiencies highlight the need for a unified strategy that integrates FDRL-driven information with energy collection to enable proactive, sustainable resource management.

Furthermore, A comprehensive analysis of security, privacy, and resource efficiency within IoT-Fog networks: A thorough analysis offers a detailed examination of the interplay between resource management and security and privacy methods in IoT-Fog systems. The findings underscore the significance of harmonizing efficiency with protection, a viewpoint that aligns with the sustainability-focused design of the EAVM protocol^37^.

Recent research, such the AoI-aware DRL framework for energy-harvesting IoT networks^38^ and the Hybrid FL–DRL model for cooperative resource allocation^39^, has enhanced the amalgamation of deep reinforcement learning and federated optimization in sustainable IoT systems.

Nonetheless, these methodologies predominantly focus on transmission regulation or model coordination instead of comprehensive infrastructure management. The proposed EAVM protocol presents a cohesive architecture that simultaneously incorporates virtualization, migration, and adaptive hybrid energy harvesting, facilitating cross-layer optimization of energy efficiency and network performance in dynamic Green IoT settings.

A comparative summary is offered in Table 1 to elucidate prior methodologies and emphasize the innovation of the proposed EAVM protocol.

**Table 1:** Comparison of related studies and the proposed EAVM protocol.

Study/Year	Technique	Energy Harvesting	Learning Mechanism	Scalability	Sustainability Focus	Main Limitation
Lightweight Virtualization Framework [25]	Static virtualization	✗	None	Low	Medium	No dynamic migration
Predictive Migration Protocol [26]	Predictive analytics	✗	Centralized	Medium	Low	High migration overhead
Federated Learning for Energy-Aware Task Allocation [28]	Distributed learning	✗	Federated Learning	High	Medium	No energy harvesting support
Deep Reinforcement Learning for VM Placement [29]	VM allocation	✗	DRL	Medium	Medium	Lacks distributed scalability
Federated DRL for IoT Traffic Offloading [30]	Resource offloading	✗	FDRL	High	Low	No energy harvesting integration
Green IoT: Energy Efficiency, Renewable Integration, and Security Implications [31]	Green IoT framework	✓ (renewable integration)	Policy-based	High	High	No virtualization and migration mechanism
Energy-Aware Virtual Resource Scaling [34]	Adaptive virtualization	✓ (limited)	Rule-based	Moderate	Medium	No FDRL integration
Hybrid FL-DRL for Resource Allocation [39]	Hybrid federated + DRL coordination	Partial	Hybrid FL-DRL	High	Medium–High	Focuses only on aggregation efficiency, lacks virtualization/migration integration
AoI-aware DRL for Energy-Harvesting IoT Networks [38]	Deep RL-based transmission control with adaptive energy management	✓ (solar/RF)	Deep Reinforcement Learning (DRL)	Moderate	High	Focused on transmission optimization; lacks virtualization and migration mechanisms
Proposed EAVM (this work)	Adaptive virtualization and migration	✓ (hybrid solar/RF)	Federated Deep Reinforcement Learning (FDRL)	High (distribute)	High	—

In conclusion, prior research has advanced in virtualization, migration, and energy harvesting within Green IoT Wireless Sensor Networks; nevertheless, it lacks a cohesive framework that adapts dynamically to user needs and environmental constraints. The proposed EAVM protocol addresses these shortcomings by combining FDRL with migration optimized for harvesting, hence enhancing efficiency and sustainability for Green IoT applications.

## Proposed energy-aware adaptive virtualization and migration protocol

This chapter provides a thorough introduction to the EAVM protocol, designed to enhance energy efficiency and extend the operational lifespan of wireless sensor networks in Green IoT environments. The EAVM protocol deviates from conventional methods that prioritize performance and response time by embracing a comprehensive strategy that integrates energy harvesting capabilities with FDRL.

The principal objective of this protocol is to provide an autonomous and intelligent system for the management of virtual resources. It effectively assesses resource allocation and migration by analyzing the present energy condition of sensor nodes. This chapter commences with an overview of the multi-layered EAVM architecture, advances to the mathematical modeling and fundamental formulas, and culminates with a detailed elucidation of the FDRL-based decision-making algorithm. This approach ensures environmental sustainability by significantly reducing energy use while maintaining a high Quality of Service (QoS) for users.

### System architecture

**Fig. 1:**
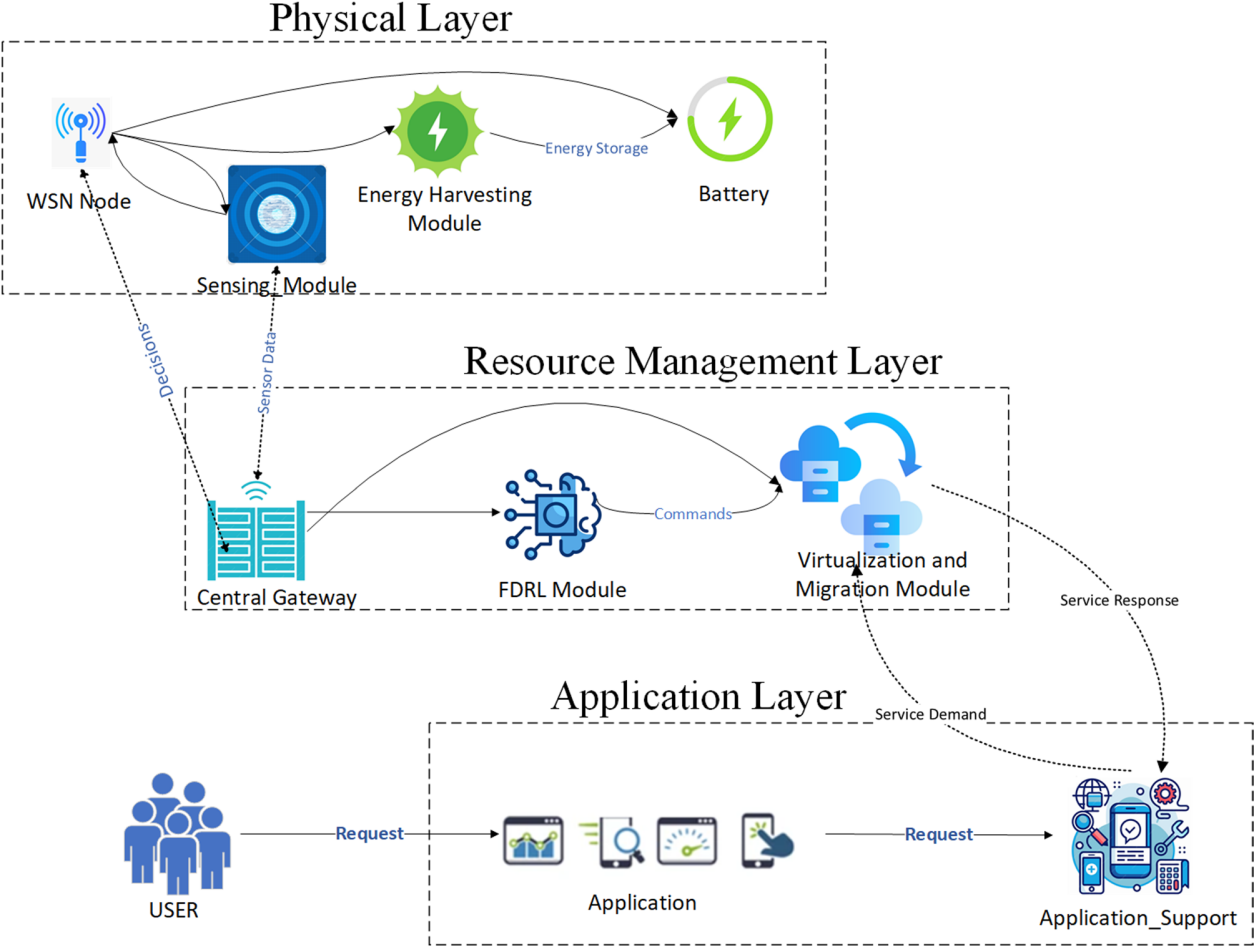
Proposed EAVM Protoco.

The EAVM protocol employs a multi-layered architecture designed to address the challenges of energy and resource management in IoT-based WSNs. This design is based on an intelligent and distributed system that employs real-time energy data to enable informed decisions regarding resource virtualization and migration. The architecture consists of three main layers: the Physical Layer, the Resource Management Layer, and the Application Layer, as seen in Fig. 1, Overall Architecture of the EAVM Protocol. The criteria for these levels are described below.

#### Physical layer

**Fig. 2:**
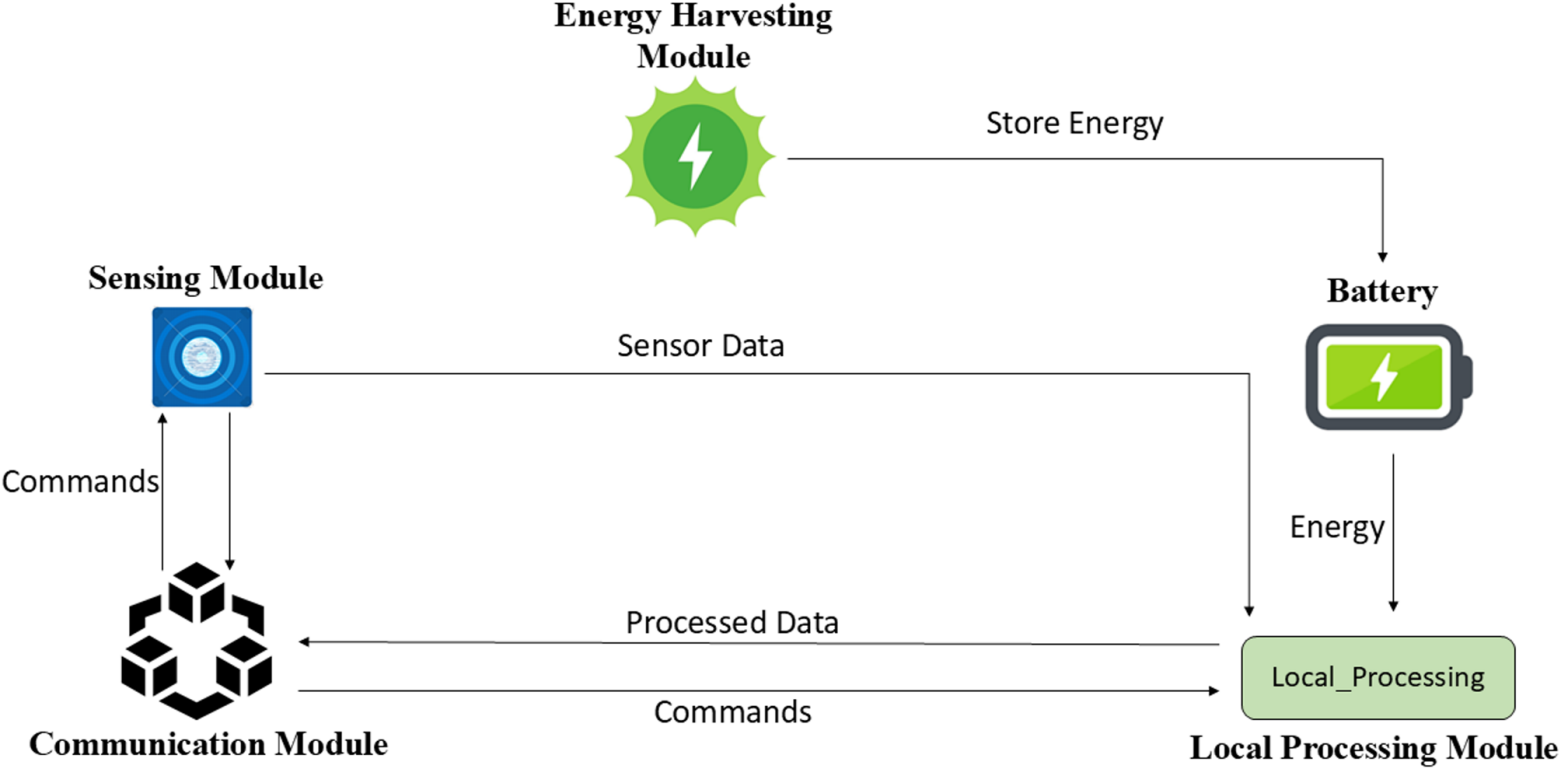
Components of an Energy-Harvesting Sensor Node.

The Physical Layer constitutes the fundamental architecture of the EAVM system, consisting of WSN nodes engineered to function as independent units. Each node is designed to gather environmental data and optimize its energy consumption, hence assuring operational sustainability within Green IoT frameworks. The architecture of a node, depicted in Fig. 2 Components of an Energy-Harvesting Sensor Node, incorporates numerous essential components to fulfill these aims.

The Sensing Module is responsible for collecting physical data regarding the environment, encompassing characteristics such as pressure, temperature, and humidity. This module ensures precise and reliable data collection, serving as the primary input for the system’s decision-making processes.

A significant advancement in the node’s architecture is the Energy Harvesting Module, which enables the conversion of ambient energy sources such as thermal, vibrational, or solar energy into usable electrical power.

This study focuses on an energy harvesting module that predominantly employs solar and RF hybrid energy sources, demonstrating efficacy in outdoor IoT settings. The solar cell generates an average power output of 120–150 mW under standard lighting circumstances, but the RF harvester adds an extra 20–30 mW from ambient communication signals. This hybrid architecture ensures uninterrupted power supply, even in low-light conditions, hence prolonging node longevity and enhancing system sustainability.

The node’s battery retains this harvested energy, significantly reducing dependence on external power sources and enhancing the node’s ability to operate independently in resource-limited environments.

The Battery Management Module supervises the battery condition of the node, tracking critical metrics such as charge level, discharge rate, and recharge rate. This module generates critical data that informs the decision-making process based on FDRL. It intermittently transmits this information to the Resource Management Layer, enabling dynamic and energy-efficient resource allocation.

The Communication Module facilitates bidirectional communication between the node and the central gateway. It is tasked with receiving operational commands from the Resource Management Layer and transmitting collected environmental data to the gateway, ensuring seamless integration within the overall network architecture.

These aspects collectively enable the Physical Layer to achieve the EAVM protocol’s objectives of energy efficiency and environmental sustainability, hence building a robust foundation for intelligent resource management in Green IoT Wireless Sensor Networks.

#### Resource management layer

**Fig. 3:**
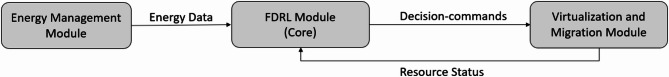
Structure of the Resource Management Layer at the Gateway.

The Resource Management Layer serves as the intellectual core of the EAVM system, either at the central gateway or an edge server. This layer is responsible for collecting and analyzing data from WSN nodes and making informed decisions to optimize resource allocation. The layout of this layer, seen in Fig. 3 Structure of the Resource Management Layer at the Gateway, comprises several critical components designed to enhance system efficiency and sustainability.

The Energy and Network Data Collection Module aggregates critical information regarding network traffic and the energy status of each node. This module ensures the availability of extensive data for real-time analysis, forming the foundation for effective resource management decisions.

The FDRL Module functions as the cognitive nucleus of the system. This module employs trained local models from distinct nodes to construct a suitable global model that guides resource allocation and virtualization strategies. The FDRL methodology enables decentralized learning of optimal management strategies while ensuring data privacy, allowing the system to adapt dynamically to changing network conditions.

The Virtualization and Migration Module executes tasks based on directives from the FDRL Module. It facilitates the creation of virtual resources on nodes with abundant energy and, when necessary, orchestrates the transfer of virtual resources from nodes with inadequate energy to those with greater energy reserves. This approach minimizes service interruptions and enhances the overall efficiency of resource distribution.

These components collectively enable the Resource Management Layer to support intelligent, energy-efficient resource allocation and migration, ensuring optimal performance and environmental sustainability in Green IoT Wireless Sensor Networks.

#### Application layer

The Application Layer serves as the interface between the EAVM system and its users, ensuring the reliable and high-quality delivery of requested services. This layer aims to facilitate seamless interaction between user requirements and the underlying resource management architecture, providing optimal performance in Green IoT WSNs.

The User Demand Management Module processes incoming user service requests and forwards them to the Resource Management Layer. This module ensures accurate collection and effective conveyance of user needs, enabling the system to respond promptly to evolving requests.

The Service Support Module is tasked with guaranteeing the requisite QoS by overseeing the distribution of resources necessary to fulfill user requirements. Additionally, it provides critical input to the Resource Management Layer, including metrics like as response time and service success rates. This feedback loop enables the continuous improvement of the FDRL model, augmenting the system’s adaptability and efficiency.

The EAVM system’s multi-tiered architecture provides a holistic solution to the challenges of Green IoT networks by integrating physical energy harvesting capabilities with decentralized artificial intelligence. This extensive technique fosters a self-regulating and sustainable system, proficient in balancing energy conservation with efficient service delivery.

### Mathematical modeling

This section delineates the mathematical modeling of the EAVM protocol, emphasizing energy and user demand management in WSNs. The modeling facilitates the FDRL-based method for optimal resource allocation and migration, emphasizing energy efficiency.

#### Energy and consumption modeling at each node

In a wireless sensor network including N sensor nodes, the energy condition of each node $\:i\left(ifrom1toN\right)$ At time t, the model is constructed by initially calculating the components of energy consumption, subsequently determining total consumption and available energy. Perceiving energy, $\:{E}_{S}$, is:

 

1 $$\:{E}_{S}\:=\:{C}_{S}\:*\:D$$

where $\:{C}_{S}$ is the energy cost per sensing sample and $\:D$ is the total number of samples collected.

The processing energy, $\:{E}_{P}$, denotes the energy required for computational tasks.

2 $$\:{E}_{P}\:=\:{C}_{P}\:*\:{I}_{P}$$

where $\:{C}_{P}$ denotes the energy cost per instruction and $\:{I}_{P}$ is the number of processing instructions executed by the node.

The communication energy, $\:{E}_{C}$, encompassing both transmission and reception, is:

3 $$\:{E}_{C}\:=\:{E}_{T}\:+\:{E}_{R}$$

where $\:{E}_{T}$ is the transmission energy consumed during data sending, and $\:{E}_{R}$ is the reception energy consumed when receiving data packets, computed as:

4 $$\:{E}_{T}=\left({E}_{EL}+{E}_{A}*{d}^{2}\right)*{L}_{P}$$

where $\:{E}_{EL}$ represents the electronic circuit energy per bit, $\:{E}_{A}$ is the amplifier energy per bit per unit distance, $\:d\:$is the transmission distance, and $\:{L}_{P}$ is the packet length, computed as follows:

5 $$\:{E}_{R}\:=\:{E}_{E\:L}*\:{L}_{P}$$

$\:{E}_{EL}$ denotes the energy consumed by the electrical circuit per bit, $\:{E}_{A}$ represents the amplifier energy per bit per unit distance, d indicates the transmission distance, and L_P_ signifies the data packet length.

Total energy consumption, $\:{E}_{CT}$, aggregates these components:

6 $$\:{E}_{CT}\:=\:{E}_{S}\:+\:{E}_{P}\:+\:{E}_{C}$$

where $\:{E}_{CT}$ denotes the total energy consumption of a node over the interval, $\:{E}_{S}$ is the sensing energy (Eq. 1), $\:{E}_{P}$ is the processing energy (Eq. 2), and $\:{E}_{C}$ is the communication energy (Eq. 3).

The available energy, $\:{E}_{A},$ denotes the energy accessible for a node.

7 $$\:{E}_{A}\:=\:{E}_{H}\:+\:{E}_{B}\:-\:{E}_{CT}$$

where $\:{E}_{H}$ refers to harvested energy and $\:{E}_{B}$ is the remaining battery energy from the previous cycle.

This methodical methodology guarantees precise energy monitoring for each node.

#### Application demand and QoS modeling

Application demand, $\:{A}_{D},$ encompasses energy efficiency and is delineated as follows:

8 $$\:{A}_{D}=f\left({U}_{C},{R}_{C},{\eta\:}_{e}\right)$$

where $\:{U}_{C}$ represents active users, $\:{R}_{C}$ denotes available resources, and $\:{{\upeta\:}}_{e}$ signifies the energy efficiency factor. The EAVM protocol seeks to optimize $\:{A}_{D}$ while reducing $\:{{\upeta\:}}_{e}$, hence facilitating effective user request management within energy limitations.

### Fdrl-based virtualization and migration algorithm

This section presents the FDRL algorithm, the intelligent core of the EAVM protocol. This technique enables the system to autonomously optimize resource allocation and virtualization without human intervention.

#### The FDRL framework

The FDRL decision-making process within the EAVM protocol is structured as a Markov Decision Process (MDP) characterized by the tuple $\:\left(S,A,P,R,{\upgamma\:}\right),$ representing states, actions, transition probabilities, rewards, and a discount factor. This facilitates autonomous resource virtualization and migration determinations.

For exemplification, take a straightforward instance. In a Green IoT network, each sensor node monitors its local battery status and workload. Through deep reinforcement learning, it determines whether to execute a task locally or transfer it to another node to conserve energy. In federated learning mode, each node trains its local model and periodically transmits only model parameters to the central gateway. The gateway consolidates these updates to create a global model that directs energy-efficient decisions for all nodes. This collaborative learning method enables EAVM to optimize energy consumption while sustaining high-quality service without the transmission of raw data.

The system’s state at time t is formally defined as follows:

9 $$\:{s}_{t}=\left({E}_{A},{A}_{D},{U}_{C},{R}_{C}\right)$$

where $\:{E}_{A}$ is the available energy of the node at time $\:t$ (Joules), $\:{A}_{D}$ denotes the application demand measured over the current decision interval (derived from Eq. 8), $\:{U}_{C}$ is the number of active user requests observed at time $\:t$, and $\:{R}_{C}$ represents the available resource capacity (CPU/memory/bandwidth aggregate). For learning stability, all state variables are min–max normalized to [0,1] before being fed to the FDRL agent.

The action space, A, comprises:

10 $$\:A=\left({a}_{m},{a}_{v},{a}_{n}\right)$$

$\:{a}_{m}$ transfers virtual resources from a low-energy node to a high-energy node, $\:{a}_{v}$ creates new virtual resources on a node with adequate energy, and $\:{a}_{n}$ indicates that no action is taken.

The transition function$\:,P\left({s}^{{\prime\:}}|s,a\right),$ characterizes state changes according to network dynamics, as acquired by the FDRL model. The reward function integrates QoS, energy efficiency, and diminished energy usage. QoS is:

11 $$\:QoS=1-{R}_{T}/Max{R}_{T}$$

$\:{R}_{T}$ denotes response time. Energy efficiency, denoted as $\:{E}_{E}$, is defined as:

12 $$\:{E}_{E}={S}_{R}/{E}_{ct}$$

where $\:{S}_{R}$ represents the total number of successfully completed requests, and $\:{E}_{ct}$ is the total energy consumption of the node obtained from Eq. (6). This ratio defines the energy efficiency of the node, expressing how effectively the consumed energy contributes to successful service delivery.

$\:{S}_{R}$ denotes successful requests. The total award is:

13 $$\:R\:=\:\alpha\:\:*\:QoS\:+\:\beta\:\:*\:{E}_{E}\:-\:\gamma\:\:*\:{E}_{ct}$$

where $\:{\upalpha\:}$, $\:{\upbeta\:}$, and $\:{\upgamma\:}$ are weighting coefficients ranging from 0 to 1 that balance the importance of service quality, energy efficiency, and energy consumption, respectively. QoS denotes the quality of service obtained from Eq. (11), EE represents the energy efficiency from Eq. (12), and $\:{E}_{ct}$ is the total energy consumed by the node. A higher reward value indicates better overall system performance with optimized energy use and service stability.

The global model is revised using federated averaging.

14 $$\:{w}_{g}lobal=su{m}_{k}\left({n}_{k}/{N}_{t}otal\right)*{w}_{k}$$

$\:{w}_{k}$ represents the local model weights, $\:{n}_{k}$ denotes the number of data samples at node k, and $\:{N}_{t}otal$ is the summation of $\:{n}_{k}$. This guarantees scalable, privacy-preserving education for enhanced resource management.

#### The FDRL learning process

**Fig. 4:**
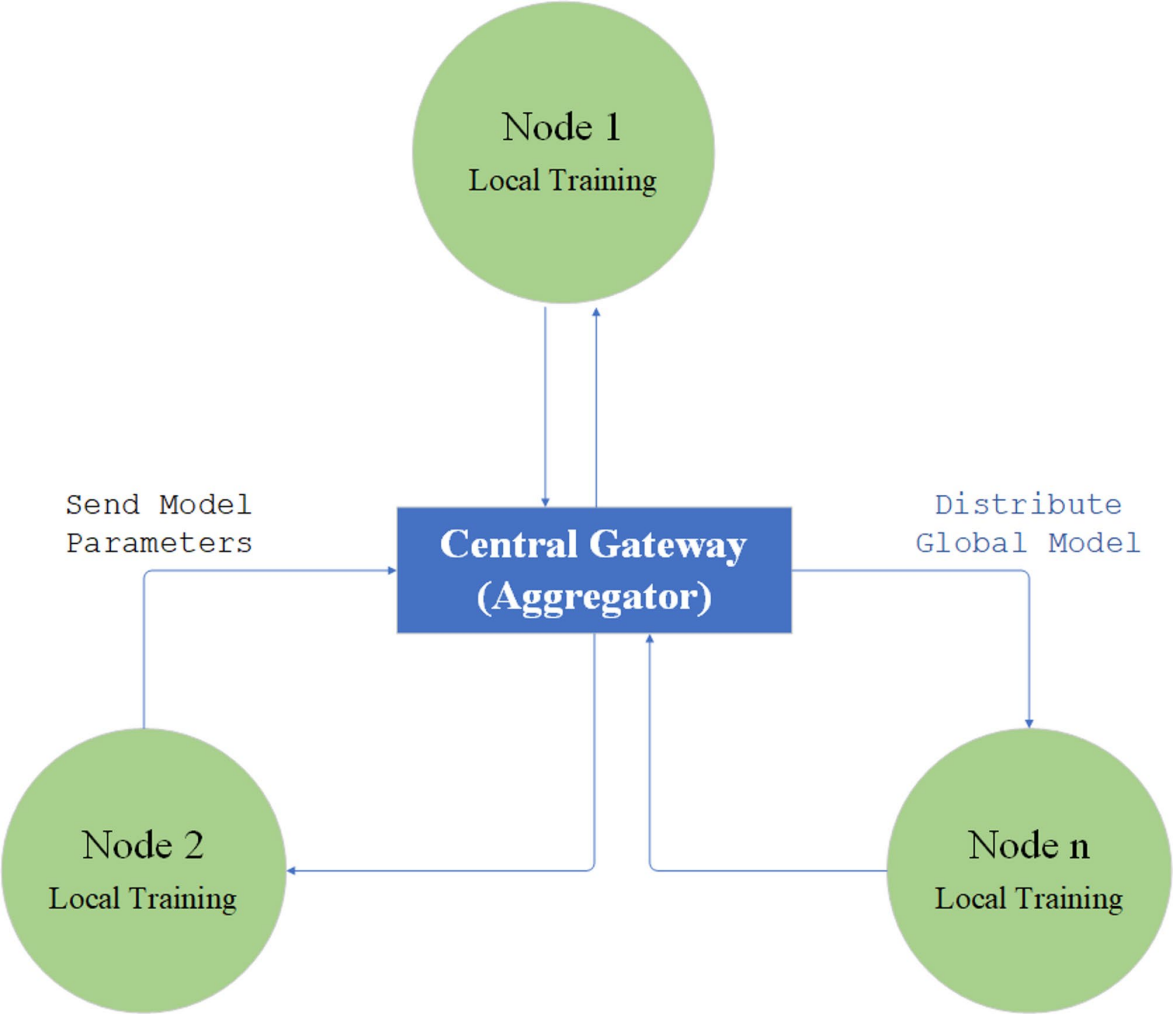
FDRL Process in EAVM.

The EAVM protocol employs an FDRL architecture to enable intelligent, distributed, and energy-efficient resource management in Green IoT Wireless Sensor Networks. Figure 4 illustrates the FDRL Process in EAVM, operating in a distributed and cyclical manner, ensuring scalability and privacy preservation while optimizing resource allocation and migration options.

The FDRL process commences with Local Training, wherein each sensor node employs its own data, including local demand and battery condition, to locally train its FDRL model. This method guarantees that raw data is retained on the node, hence reducing privacy threats. In the Parameter Transmission phase, each node transmits solely the model parameters to the central gateway, thereby circumventing the transfer of sensitive raw data. This safeguards data privacy while facilitating collaborative learning throughout the network.

During the Aggregation and Optimization phase, the central gateway serves as the aggregator, gathering model parameters from all nodes to formulate an ideal global model. This global model incorporates local insights to improve decision-making precision. Ultimately, in the Global Model Distribution phase, the refined global model is disseminated to all nodes, allowing them to employ it for local decision-making, therefore concluding the learning cycle.

**Fig. 5:**
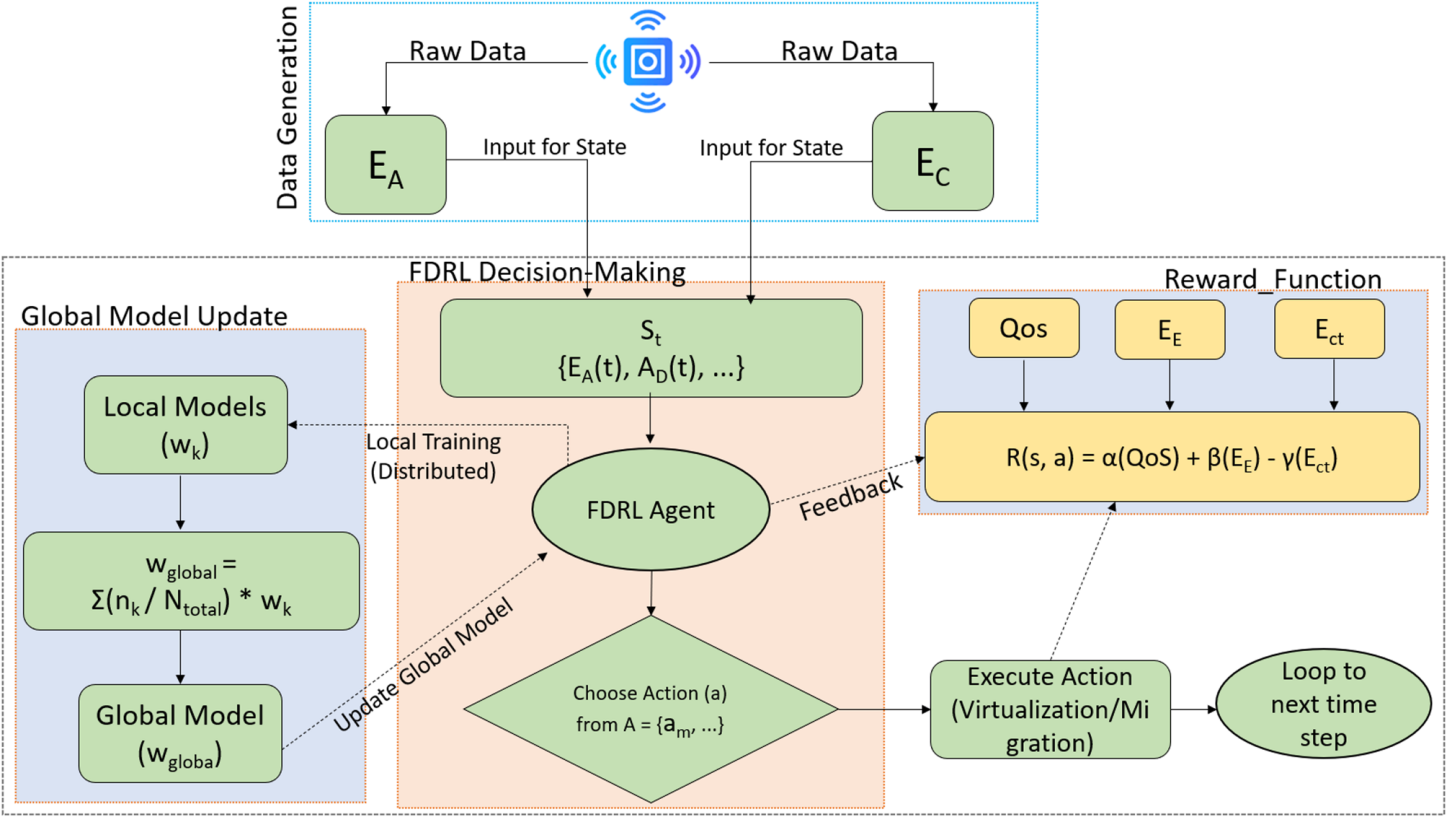
Integrated Protocol Process Flowchart.

Figure 5 Integrated Protocol Process Flowchart elucidates the relationship between the FDRL framework and the mathematical models of the EAVM protocol, outlining a continuous cycle aimed at establishing a self-organizing and energy-efficient system. The process initiates with Data Generation at the Physical Layer, wherein sensor nodes gather raw data utilized to calculate energy metrics, including $\:\left({E}_{A}\right)$ and $\:\left({E}_{C}\right).$.

The metrics, in conjunction with network parameters such as $\:\left({A}_{D}\right)$ and $\:\left({R}_{C}\right)$, constitute the State Input, denoted by the $\:\left({s}_{t}\right),$ which functions as the principal input for the FDRL model.

During the Action Selection phase, the FDRL model assesses the state vector and identifies an optimal action $\:\left(a\right)$ from the established action space, directing resource virtualization or migration choices. The results of these activities are evaluated in the Reward Calculation and Feedback phase, where performance indicators, such as QoS and $\:\left({E}_{E}\right)$, are quantified and integrated into the reward function $\:\left(R\left(s,a\right)\right).$ This incentive offers critical input, allowing the FDRL model to enhance its decision-making abilities progressively.

The cycle culminates with the Global Model Update, during which local FDRL models from each node are routinely consolidated at the central gateway. The federated averaging formula amalgamates the local model weights to generate an optimal global model $\:\left({w}_{global}\right),$ which is subsequently redistributed to all nodes, so completing the learning cycle. This integrated methodology guarantees that resource management decisions are based on a thorough, data-driven comprehension of the network’s condition, enabling proactive and sustainable resource allocation in Green IoT Wireless Sensor Networks.

Despite federated learning maintaining data localization on individual nodes, the transmission of model parameters may potentially reveal sensitive information. The EAVM framework incorporates safe parameter aggregation and lightweight encryption for model synchronization. Parameters are encrypted with symmetric keys prior to transmission and aggregated at the gateway through a privacy-preserving averaging method, guaranteeing that changes from individual nodes remain indistinguishable. This solution safeguards privacy while ensuring little computing overhead in the distributed learning process.

### Energy-aware resource allocation and migration process

**Fig. 6:**
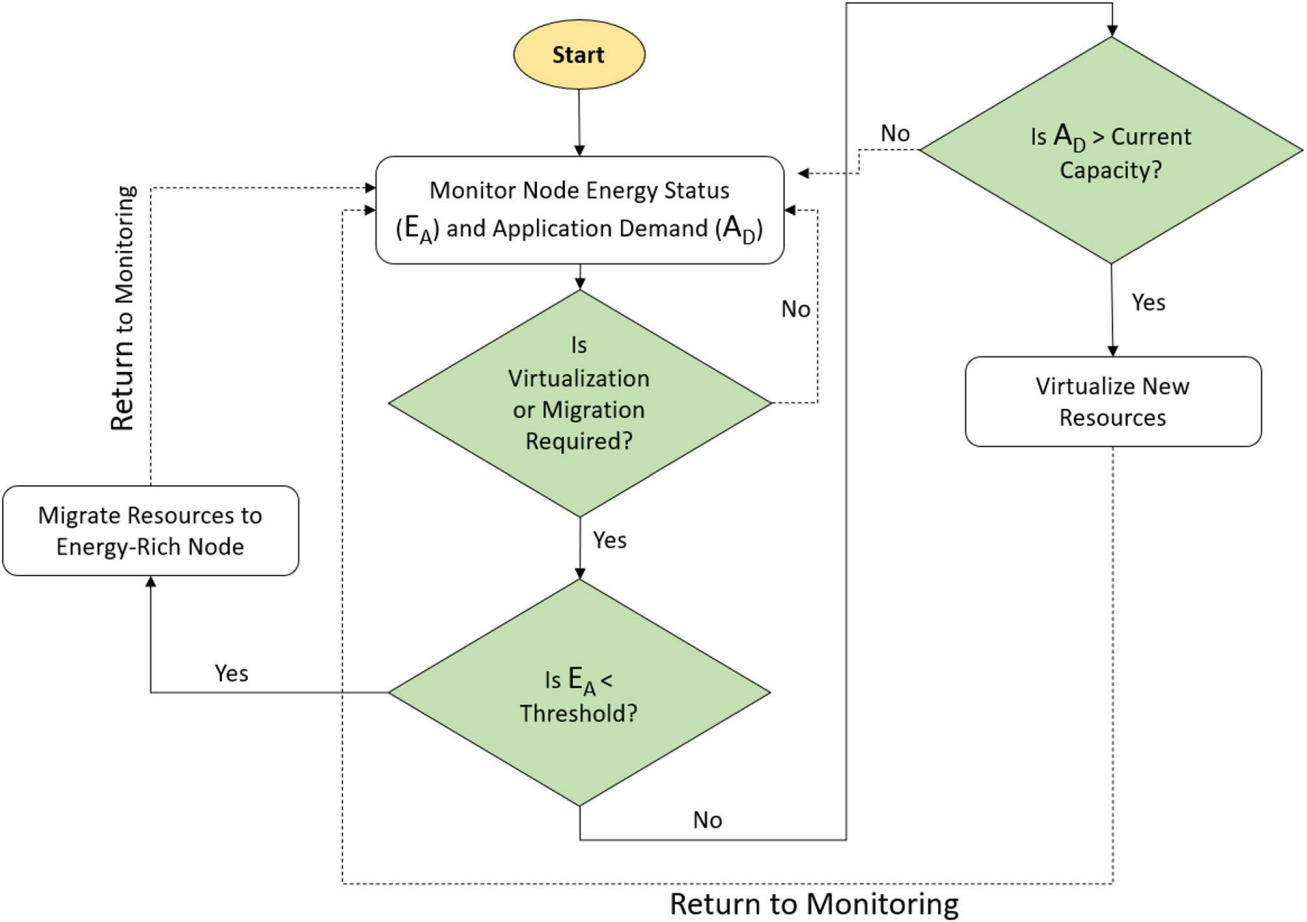
Energy-Aware Virtualization and Migration Flowchart.

The EAVM protocol utilizes a systematic algorithm to enhance resource allocation and migration in Green IoT Wireless Sensor Networks. The operating logic of this algorithm is illustrated in a detailed flowchart, as seen in Fig. 6 Energy-Aware Virtualization and Migration Algorithm. The algorithm functions through a series of clearly delineated processes, guaranteeing energy efficiency and sustainable resource management while upholding high quality of service.

The process commences with Initialization, during which the EAVM protocol is activated to begin operations. During the Monitoring phase, the system incessantly observes the energy status $\:\left({E}_{A}\right)$ and application demand $\:\left({A}_{D}\right)$ of each node, delivering real-time data to guide subsequent decisions.

In the Decision-Making phase, the FDRL model assesses the gathered data to ascertain the necessity of a virtualization or migration step. This assessment utilizes the FDRL framework to guarantee intelligent and adaptive decision-making.

During the Migration Check phase, the system evaluates if the energy status $\:\left({E}_{A}\right)$ of a node is below a specified threshold. Consequently, resource migration commences from the low-energy node to a node with adequate energy reserves, thereby averting service disruptions and preserving network stability.

The migration process in EAVM is initiated when a node’s available energy $\:{E}_{A}$ drops below a specified threshold $\:{E}_{th}$, usually established between 10% and 15% of its total energy capacity. The FDRL model incessantly observes this value in conjunction with the node’s workload intensity. When both criteria are satisfied—low energy EA < Eth and active workload demand—the model forecasts the ideal destination node characterized by elevated energy and reduced delay. The decision is bolstered by reward feedback, wherein successful migrations that diminish energy imbalance and uphold Quality of Service (QoS) provide greater incentives. This adaptive approach guarantees proactive migration instead of reactive responses to node failure.

During the Virtualization Check step, the system assesses whether the application demand $\:\left({A}_{D}\right)$ surpasses the existing resource capacity. When demand exceeds available resources, additional virtual resources are generated on nodes with sufficient energy, hence facilitating the effective management of user requests.

Ultimately, in the Return to Monitoring phase, the system resumes monitoring network conditions following the execution of requisite virtualization or migration operations. This iterative technique guarantees ongoing evaluation, facilitating anticipatory and energy-conscious resource management.

This structured algorithm, based on the FDRL paradigm, enables dynamic and sustainable resource allocation, enhancing the performance of Green IoT WSNs while reducing energy consumption and environmental effect.

## Results and discussion

This chapter provides a comprehensive evaluation and analysis of the proposed EAVM protocol. Comprehensive simulations assess EAVM’s performance, and a comparison with the most sophisticated protocols in the literature demonstrates its superiority. In dynamic Green IoT contexts, the findings are articulated to highlight the primary methods by which the proposed protocol enhances energy efficiency, service quality, and environmental sustainability. This section provides quantitative evidence to support the study’s claims and validate the efficacy of our integrated energy harvesting and FDRL architecture.

### Simulation environment

The simulations were carried out using the Contiki Cooja simulator, selected for its precision in modeling wireless sensor networks and realistic energy consumption behavior. This platform provides a reliable foundation for evaluating adaptive and energy-aware mechanisms within Green IoT systems.

The simulated network consisted of 250 wireless sensor nodes randomly deployed over the target area. Each node was equipped with an energy-harvesting unit capable of collecting hybrid solar and RF energy, with an overall harvesting efficiency ranging from 0.6 to 0.8. Nodes were initialized with 50 Joules (J) of energy, and the EAVM protocol dynamically activated its adaptive control process when a node’s residual energy fell below 5 J or when sufficient harvested energy became available to support migration and virtualization activities.

To emulate realistic traffic dynamics, 200 users generated between 100 and 1500 service requests at variable intervals between 30 and 180 s. These requests were handled by 10 distributed resource servers, each capable of processing 70–80 requests per time slot. Additionally, 20 fixed intermediate gateways facilitated multi-hop communication and load balancing among nodes and servers, ensuring efficient data routing across the network.

The evaluation compared the performance of the proposed EAVM protocol with six established and learning-based frameworks: the Predictive Migration Protocol (PMP)^21^, Lightweight Virtualization Framework (LVF)^20^, F-SVNE^32^, Energy-Harvesting-Aware FDRL (AOI-AWARE DRL)^34^, and Hybrid FL–DRL^33^. All models were simulated under identical network parameters and environmental conditions. For frameworks without explicit virtualization or migration procedures, equivalent functional parameters were defined to measure their effective resource allocation and reconfiguration performance, ensuring a fair and consistent comparison.

### Performance metrics

The performance of the EAVM protocol was evaluated through a comprehensive set of quantitative metrics designed to measure its efficiency, responsiveness, and sustainability within Green IoT environments. All metrics were uniformly applied across the six compared frameworks under identical simulation settings to ensure analytical consistency and fairness.

The Application Response Rate represents the percentage of user requests successfully processed and acknowledged during the simulation. It reflects the protocol’s ability to maintain reliable service delivery under dynamic traffic and network conditions.

The Virtualization Rate quantifies the number of new virtual resources instantiated over time as user demand increases. This metric demonstrates the protocol’s flexibility in adapting resource allocation to fluctuating workloads.

The Migration Ratio represents the proportion of virtual resource transfers that occur during system operation. A lower migration ratio indicates greater system stability and more efficient resource management, as fewer migrations are needed to maintain performance.

The Response Time measures the average delay between a user request submission and the final response delivery. This metric assesses the protocol’s capacity to minimize latency and enhance service responsiveness.

The Resource Utilization captures the average percentage of computational resources (CPU and memory) actively engaged in task processing. It provides insight into the protocol’s capability to maximize available capacity and reduce resource idleness.

Alongside these operational KPIs, two sustainability-focused measures were evaluated. The Total Energy Consumption represents the aggregate power utilization of all nodes during the simulation, serving as a direct measure of energy efficiency. The Network Lifetime denotes the operational span of the network until the initial node depletion, signifying the system’s long-term sustainability under continuous operation. Collectively, these performance measures constitute a comprehensive evaluation framework to examine EAVM’s proficiency in attaining energy-efficient, responsive, and sustainable resource management inside Green IoT-based Wireless Sensor Networks.

### Comparative analysis and discussion

This section presents a comparative analysis of the proposed EAVM protocol with existing conventional and learning-based frameworks. The following subsections discuss the simulation results obtained under identical network conditions to evaluate the protocols’ performance across multiple dimensions, including application responsiveness, virtualization efficiency, migration stability, and resource utilization. Through this analysis, the practical advantages and sustainability impact of EAVM are systematically examined and contrasted with other methods.

#### Application response

This section assesses the application response rate of the proposed EAVM protocol under diverse network settings, encompassing variable quantities of wireless sensor nodes and degrees of user demand.

**Fig. 7:**
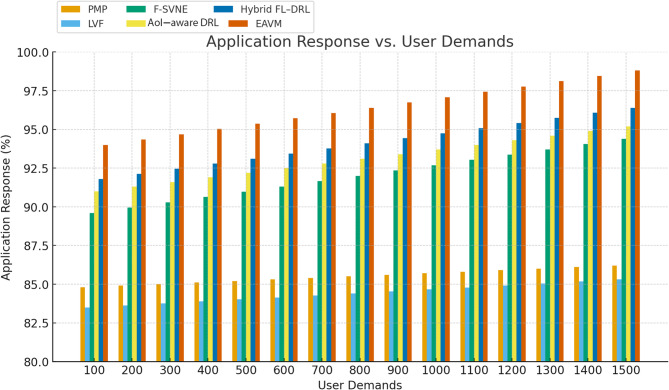
Application Response vs. User Demands.

Figure 7 illustrates that the EAVM protocol consistently attains the highest response rate under varying user demands across all load circumstances. The adaptive resource virtualization and migration techniques dynamically distribute workloads among energy-rich nodes, guaranteeing robust service during high traffic conditions.

Conversely, conventional frameworks like PMP and LVF exhibit a swift deterioration in responsiveness as user demands escalate, attributable to their static allocation algorithms and absence of predictive adaptation. The learning-based frameworks, AOI-AWARE DRL and Hybrid FL–DRL, provide superior scalability compared to traditional approaches; still, their performance is marginally inferior than EAVM, which advantages from the amalgamation of decision-making in virtualization and hybrid energy harvesting.

**Fig. 8:**
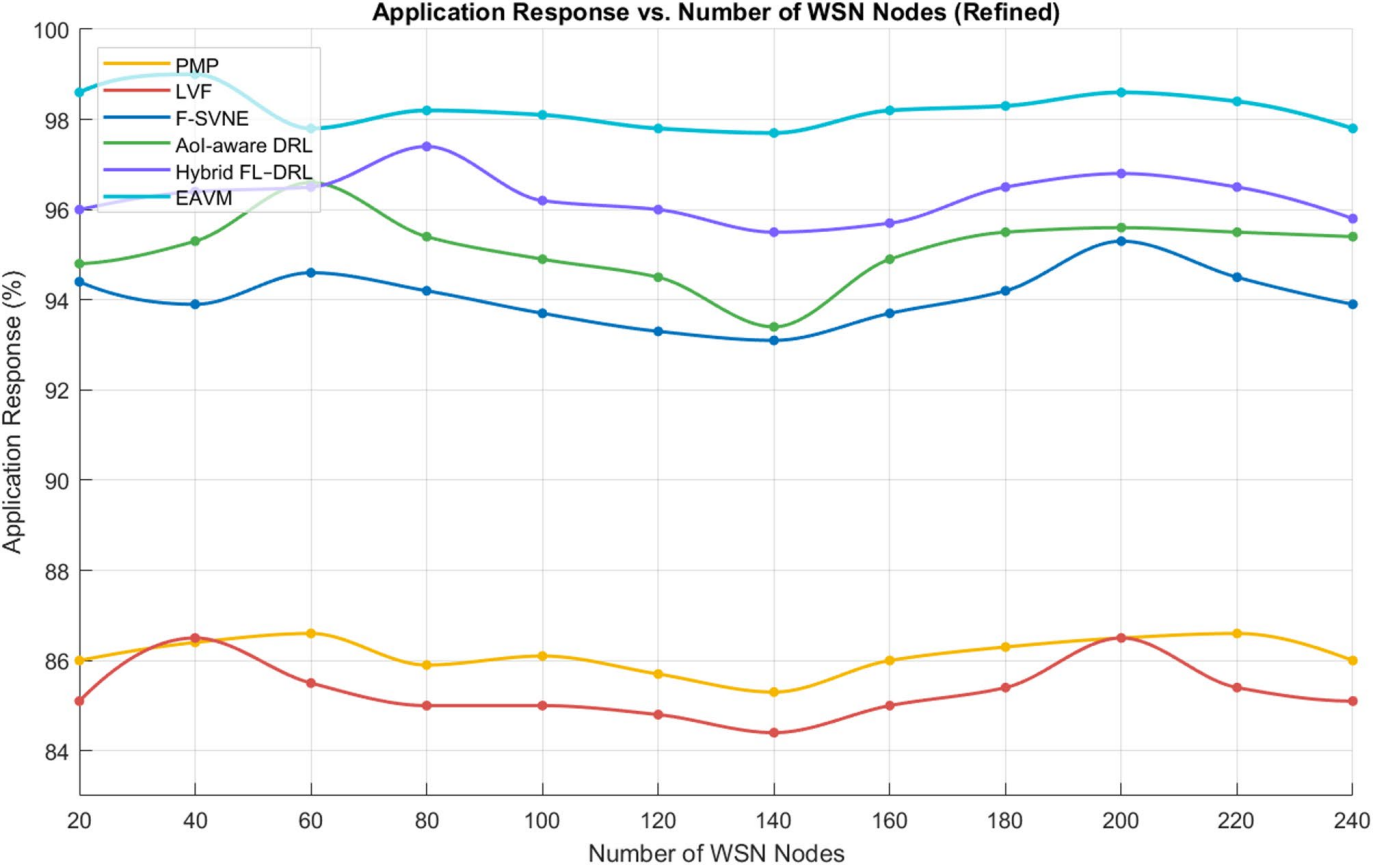
Application Response vs. Number of WSN Nodes.

Figure 8 illustrates the application response rate relative to the quantity of WSN nodes. The findings validate that EAVM exhibits exceptional performance across all network dimensions. With the growth in node density, EAVM efficiently integrates newly accessible nodes into the resource pool, utilizing their excess energy and computing capabilities.

The Hybrid FL–DRL and AOI-AWARE DRL models exhibit moderate enhancements compared to the F-SVNE baseline, however they lack the comprehensive coordination found in EAVM. As a result, EAVM attains a more equitable allocation of workloads, reducing congestion and sustaining a continually elevated response rate.

The EAVM protocol improves application responsiveness through the integration of adaptive resource management and energy awareness. It ensures consistent service delivery amidst fluctuating demand and diverse energy supply, surpassing both traditional and learning-based alternatives. These findings confirm EAVM’s capabilities to guarantee dependable and effective task execution in extensive Green IoT settings.

#### Virtualization rate

**Fig. 9:**
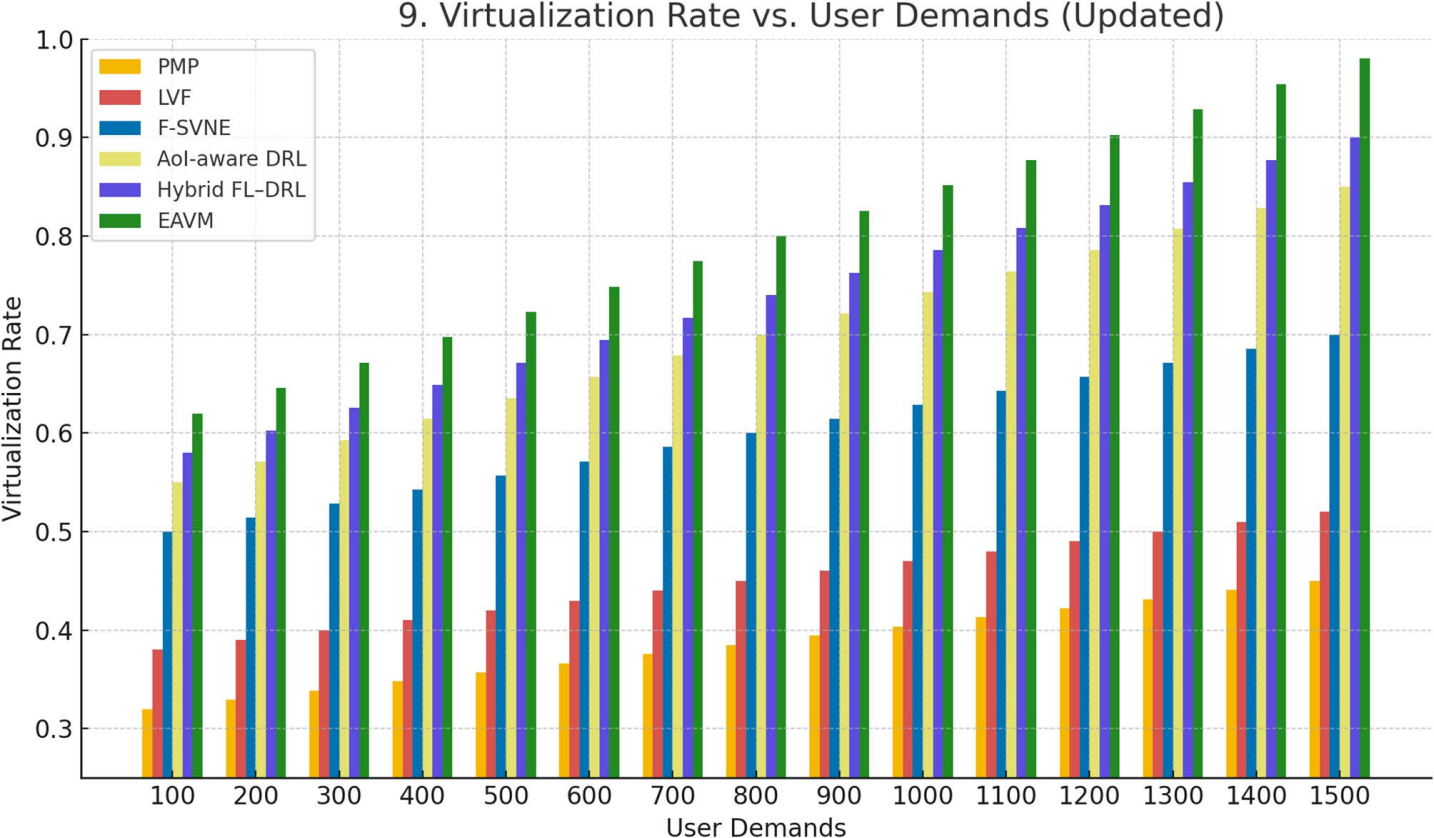
Virtualization Rate vs. User Demands.

**Fig. 10:**
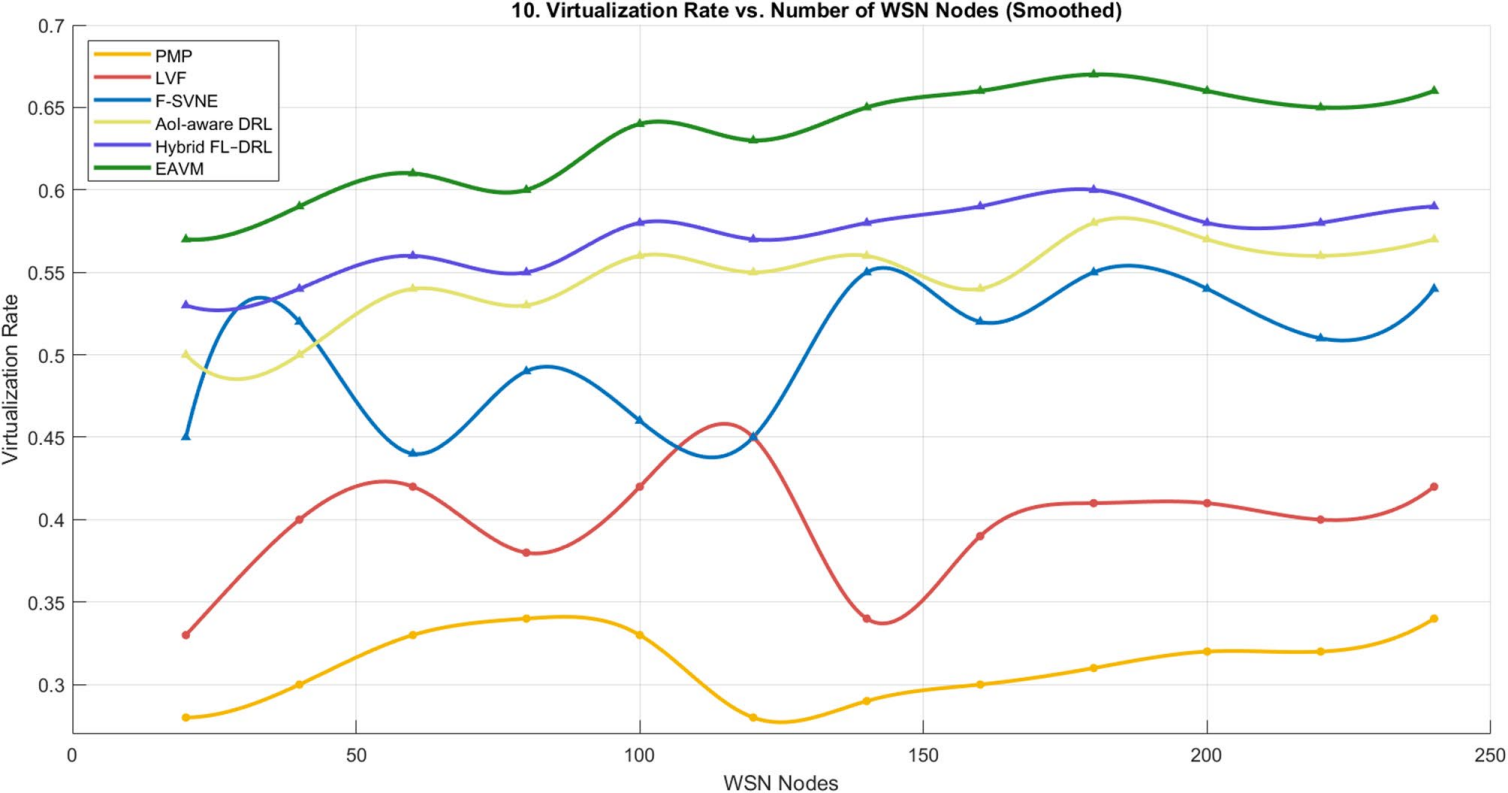
Virtualization Rate vs. Number of WSN Nodes.

The virtualization rate is a key performance metric that indicates a protocol’s capacity to dynamically allocate and manage virtual resources according to fluctuating workload requirements in Green IoT settings. Figures 9 and 10 present a comparative analysis of the proposed EAVM protocol against five benchmark frameworks: PMP, LVF, F-SVNE, AoI-aware DRL, and Hybrid FL–DRL.

Figure 9 illustrates that the EAVM protocol sustains the highest virtualization rate at all levels of user demand. Traditional frameworks like PMP and LVF show restricted scalability and adaptability, while F-SVNE offers moderate enhancements via resource prediction. In contrast, the learning-based approaches AoI-aware DRL and Hybrid FL–DRL exhibit greater adaptability but are less effective in intricate, energy-constrained IoT environments. EAVM’s federated deep reinforcement learning system facilitates anticipatory workload variation predictions and astute resource redistribution among energy-harvesting nodes. This proactive approach reduces virtual machine inactivity and guarantees consistent performance during high user traffic.

igure 10 illustrates the virtualization rate across different quantities of WSN nodes. EAVM continuously attains exceptional scalability, ensuring seamless and stable expansion with increasing node density. By including energy harvesting into the virtualization process, EAVM effectively reallocates computational loads across newly accessible nodes, therefore reducing both underutilization and migration overhead. Conversely, AoI-aware DRL and Hybrid FL–DRL, while proficient in optimizing transmission and learning, exhibit insufficient control over virtualization and migration, hence constraining their overall system efficiency.

The findings indicate that EAVM outperforms both traditional and cutting-edge models by attaining the maximum virtualization rate, optimizing resource consumption, and ensuring balanced workload allocation. This illustrates the protocol’s resilience and appropriateness for extensive Green IoT wireless sensor networks.

#### Migration ratio

**Fig. 11:**
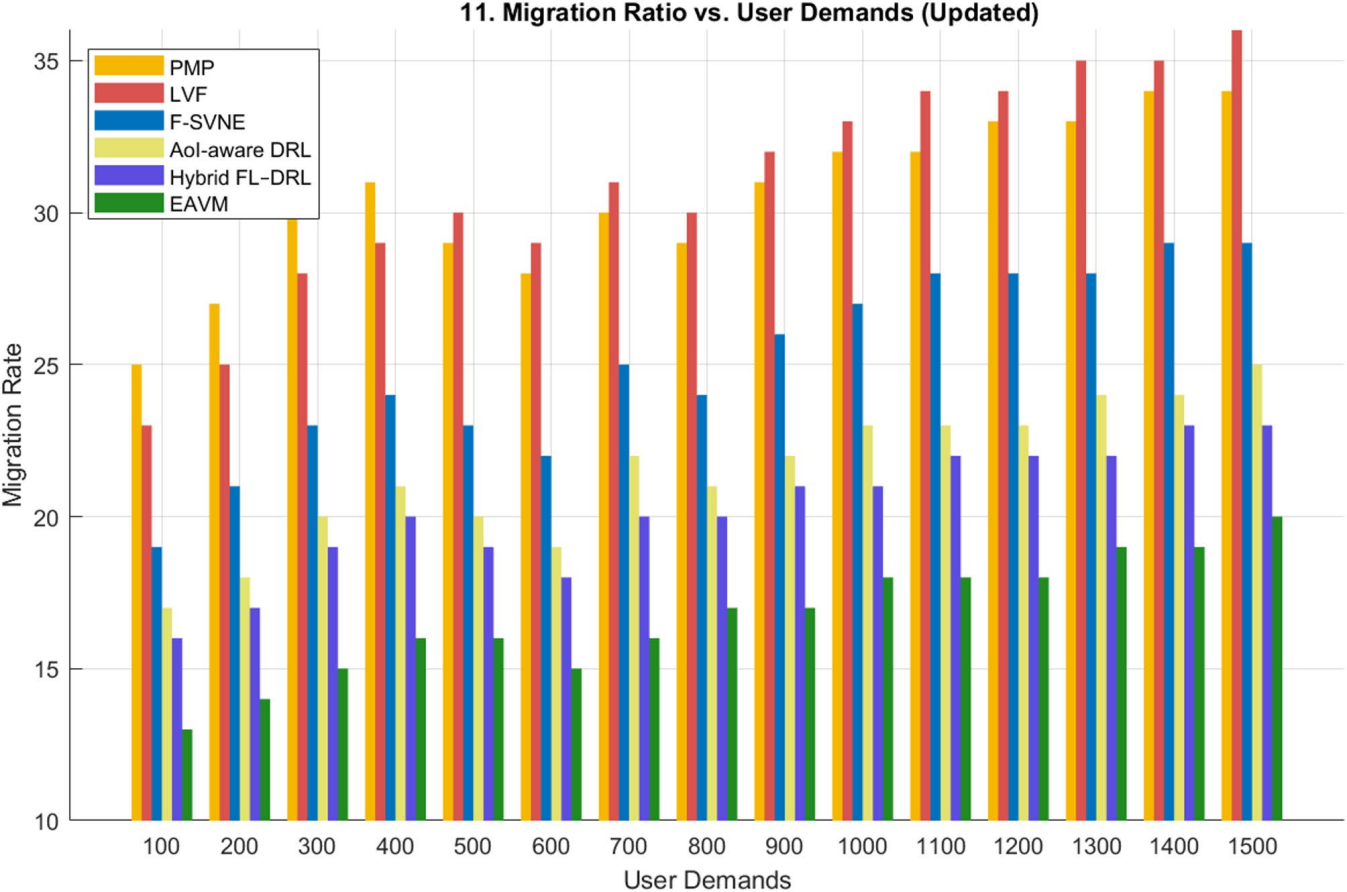
Migration Ratio vs. User Demands.

**Fig. 12:**
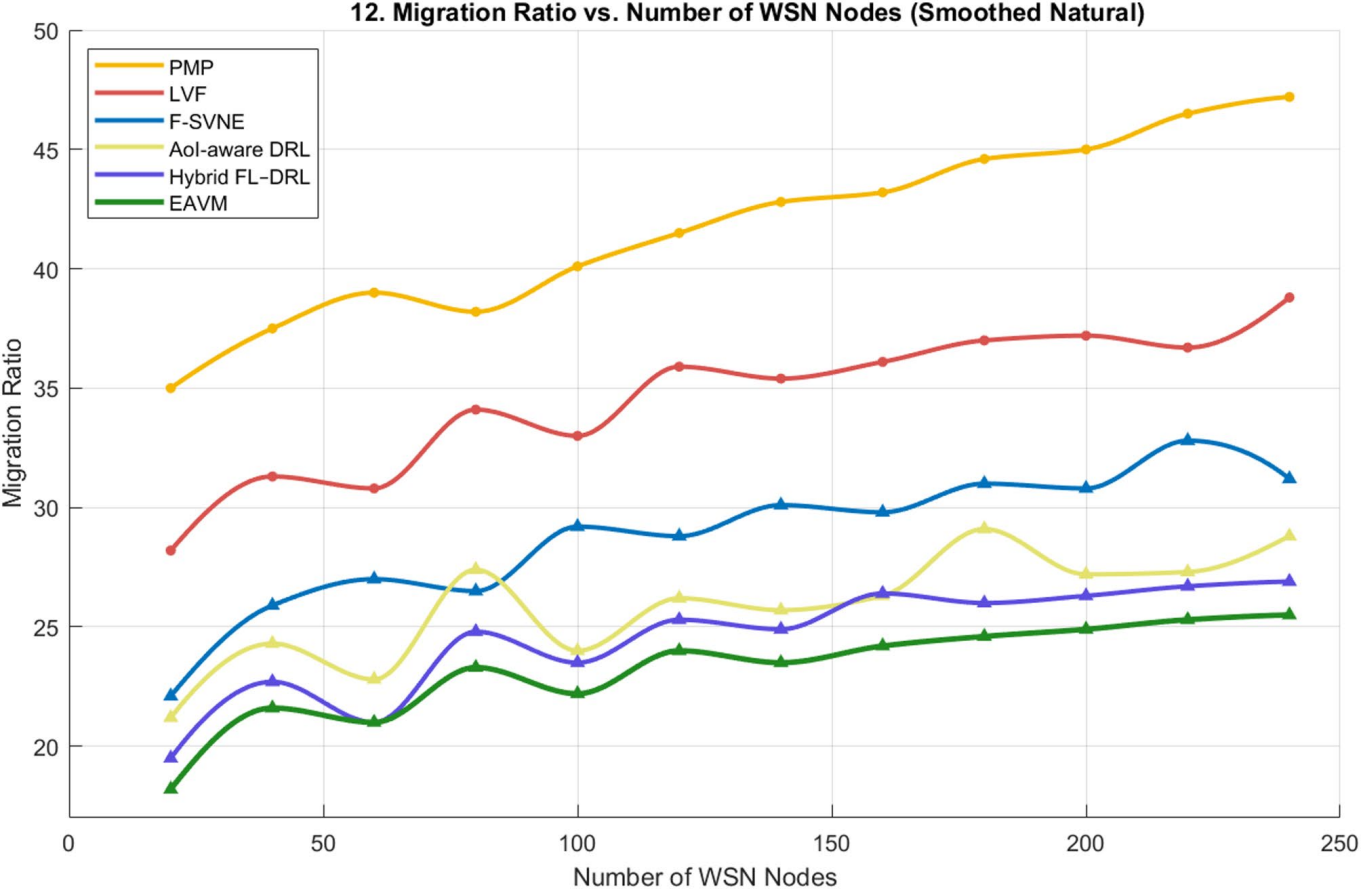
Migration Ratio vs. Number of WSN Nodes.

The migration ratio is a vital metric for assessing system stability and resource management efficacy in dynamic Green IoT networks. A diminished migration ratio indicates that the protocol may allocate resources more efficiently, decreasing superfluous task transfers and minimizing network overhead. Figures 11 and 12 depict the migration behavior of the proposed EAVM protocol in comparison to traditional and learning-based frameworks across two distinct evaluation scenarios: fluctuating user demands and variable WSN node densities.

Figure 11, which analyzes the migration ratio in relation to user demands, demonstrates that the proposed EAVM protocol sustains the lowest migration rate across all workloads. Despite a surge in user requests, EAVM demonstrates only a modest increase in migration activity, while the PMP and LVF protocols display significant escalations owing to their reactive and static allocation methodologies. The F-SVNE framework enhances stability by partial virtualization awareness; yet, it responds tardily to energy variations. The recently implemented AoI-aware DRL and Hybrid FL–DRL methodologies demonstrate enhanced adaptability through the integration of reinforcement-learning-based scheduling; nonetheless, they are deficient in explicit virtualization and migration coordination. EAVM surpasses them by concurrently handling virtualization, migration, and hybrid energy harvesting, resulting in the most balanced and stable system response.

Figure 12 illustrates the migration ratio corresponding to the growth in the number of WSN nodes. Consistent with the prior example, EAVM continuously attains the lowest migration ratio, ensuring seamless scalability across varying network sizes. The findings validate that EAVM’s federated deep reinforcement learning system anticipates workload fluctuations and reallocates resources prior to congestion or energy exhaustion. Conversely, traditional approaches (PMP, LVF) produce elevated migration rates as their migration decisions are activated subsequent to node overload. The hybrid learning methodologies considerably decrease migration frequency; however, their deficiency in cross-layer energy-aware coordination constrains their overall efficacy.

Both data indicate that the proposed EAVM protocol decreases migration overhead by roughly 5% in comparison to the nearest contemporary rival (Hybrid FL–DRL). This finding confirms its enhanced predictive decision-making and energy-efficient resource management abilities, leading to improved stability and extended network longevity in Green IoT wireless sensor settings.

#### Response time

**Fig. 13:**
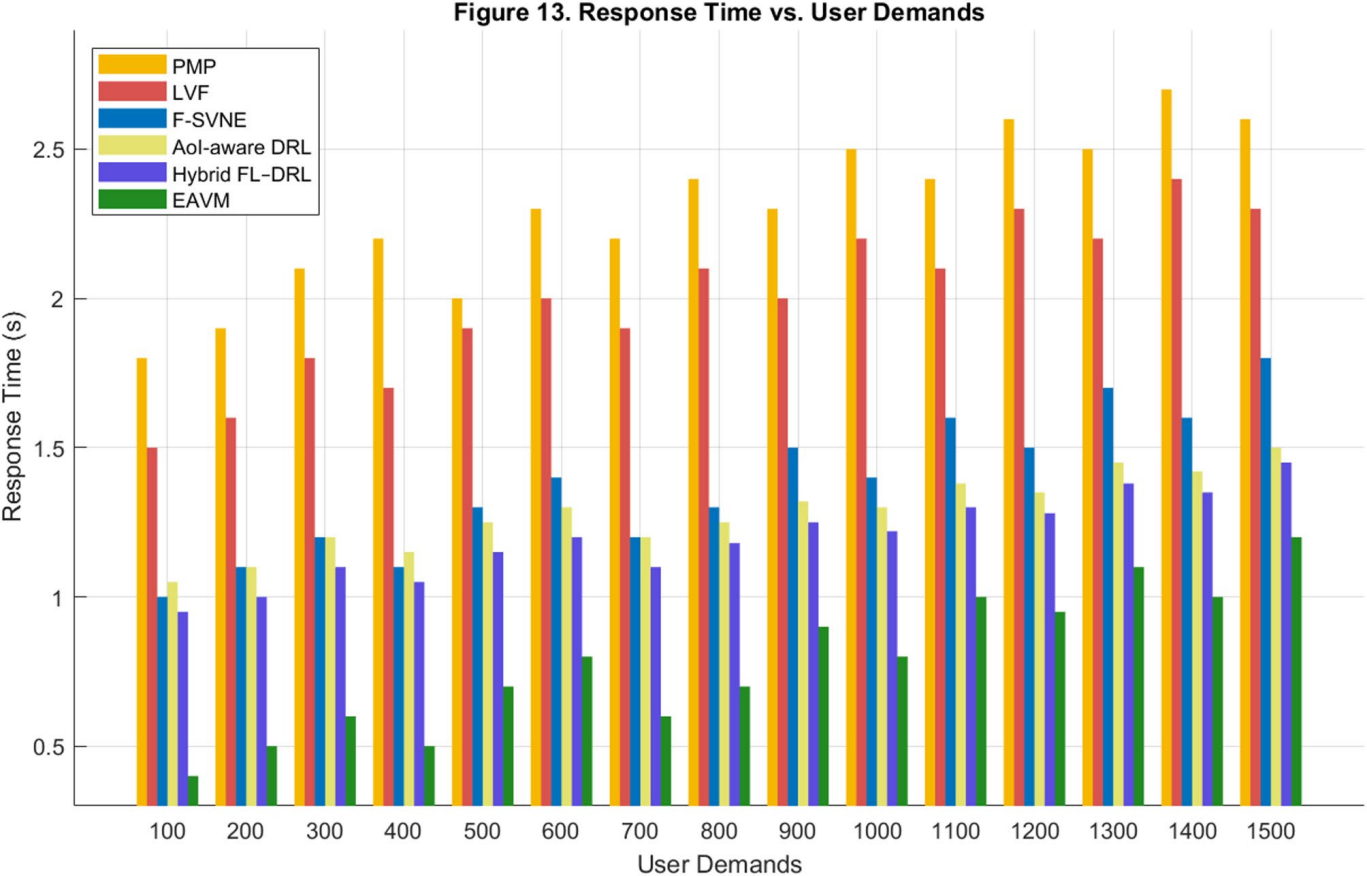
Response Time vs. User Demands.

**Fig. 14:**
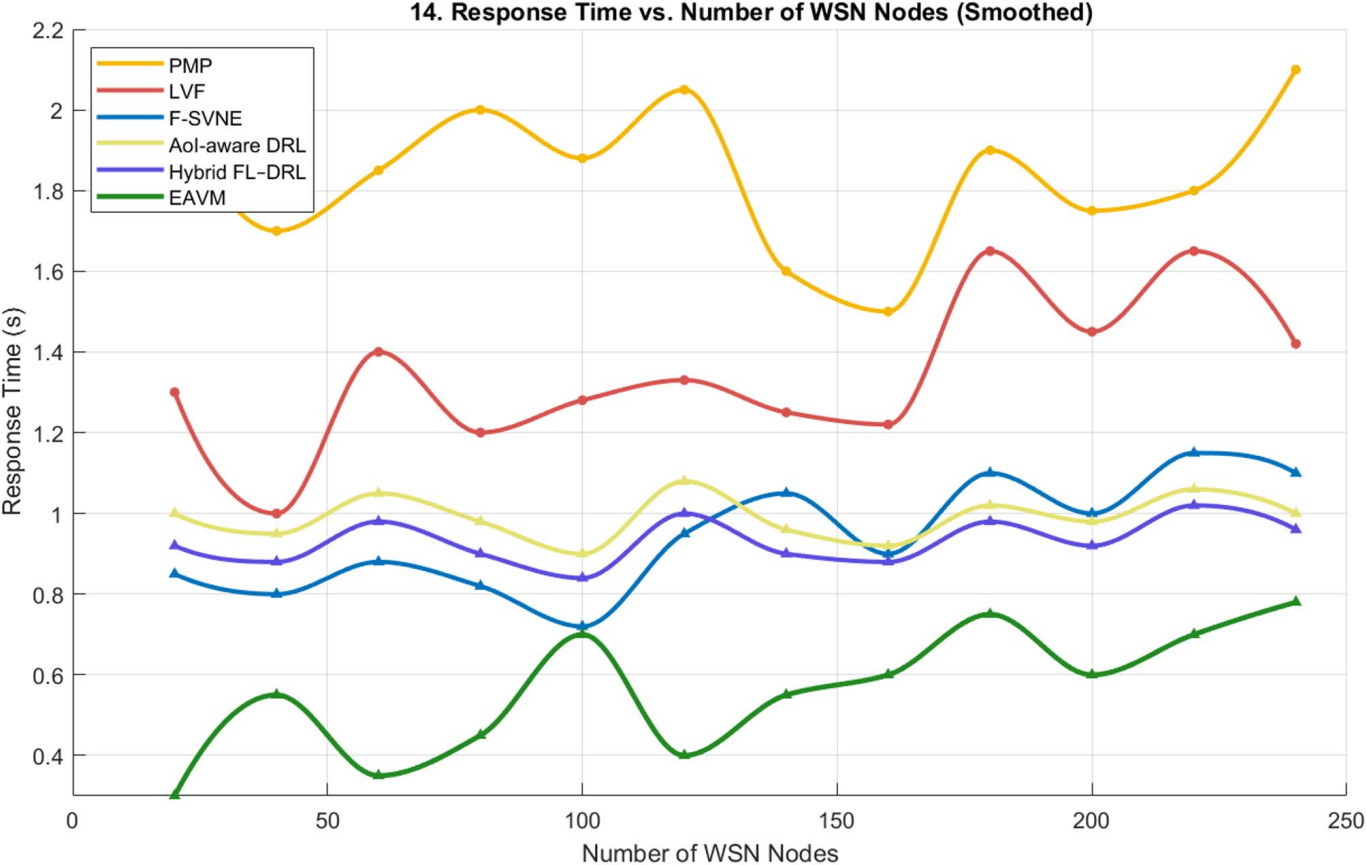
Response Time vs. Number of WSN Nodes.

Response time denotes the average interval necessary to process and fulfill a user request, serving as a primary indication of system responsiveness and service quality in Green IoT contexts. Figures 13 and 14 depict the comparative outcomes for response time over diverse user demands and changing density of WSN nodes.

Figure 13 illustrates that the proposed EAVM protocol attains the minimal response time under all workloads while evaluating the impact of escalating user demands. Despite substantial request loads, EAVM sustains consistently low latency because to its predictive scheduling method, which assigns virtual resources to energy-rich nodes prior to congestion.

Traditional methodologies (PMP and LVF) demonstrate considerable increases in reaction time when user demands escalate, owing to their reactive task management. The F-SVNE technique exhibits moderate improvement via lightweight virtualization, whereas the more recent AoI-aware DRL and Hybrid FL–DRL frameworks leverage learning-based optimization but are hindered by restricted cross-layer coordination. EAVM outperforms all these solutions by concurrently optimizing virtualization, migration, and energy harvesting procedures, facilitating more equitable resource allocation and diminished service latency.

Figure 14 illustrates the correlation between response time and the quantity of WSN nodes. As the network density increases, EAVM consistently demonstrates the lowest latency and the most seamless trend, signifying enhanced scalability and workload flexibility.

Competing protocols (PMP, LVF, F-SVNE) exhibit variable and elevated latency attributable to their centralized decision-making processes, which become inefficient in expansive networks. The learning-based AoI-aware DRL and Hybrid FL–DRL methodologies somewhat alleviate latency; nonetheless, EAVM’s federated learning framework offers expedited convergence and more energy-efficient judgments.

The EAVM protocol decreases average response time by roughly 6 to 7% relative to Hybrid FL–DRL and approximately 40% compared to traditional methods, thereby validating its capacity to guarantee real-time Quality of Service and uphold system stability amid fluctuating workload situations.

#### Resource utilization

**Fig. 15:**
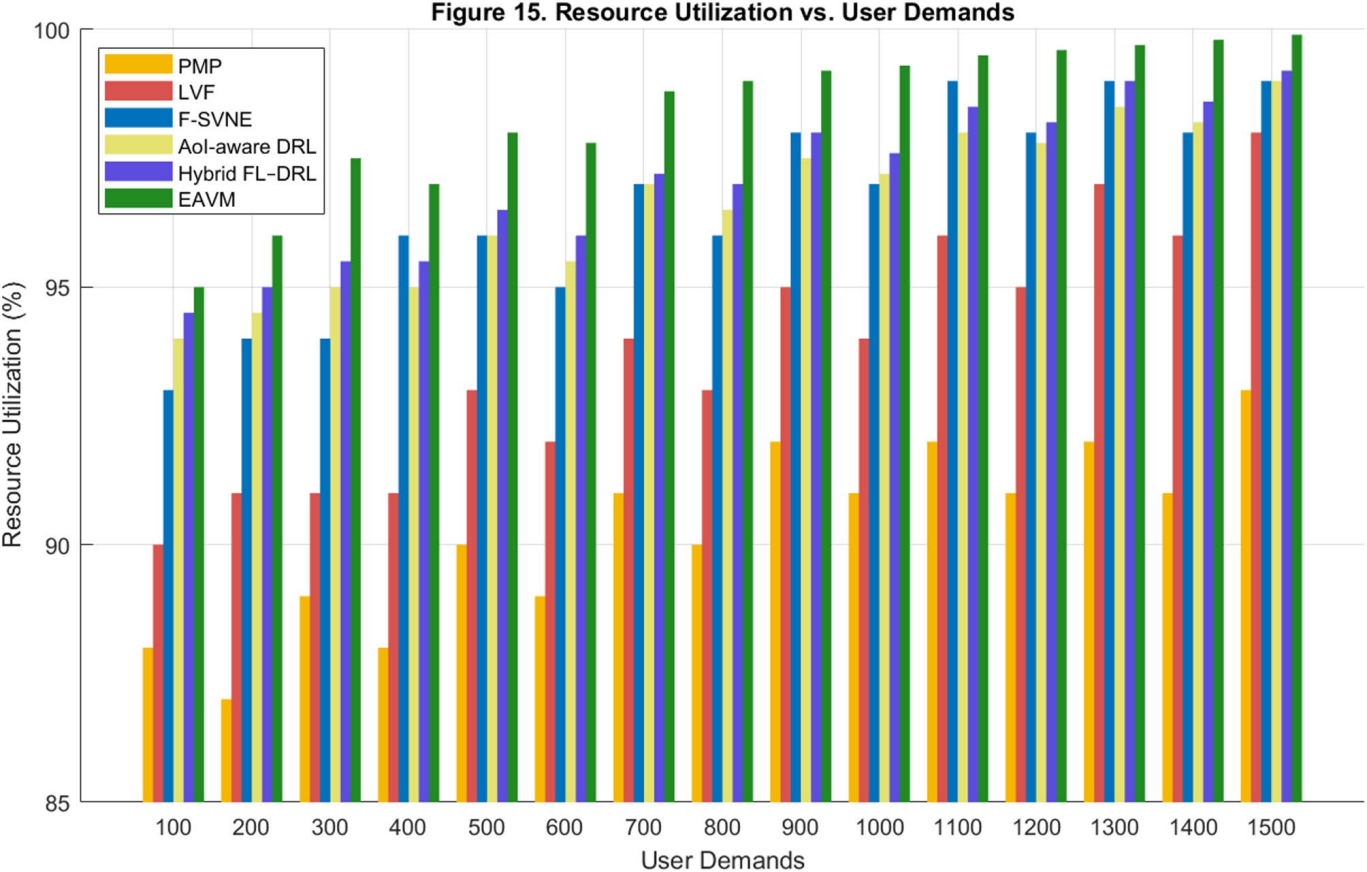
Resource Utilization vs. User Demands.

**Fig. 16:**
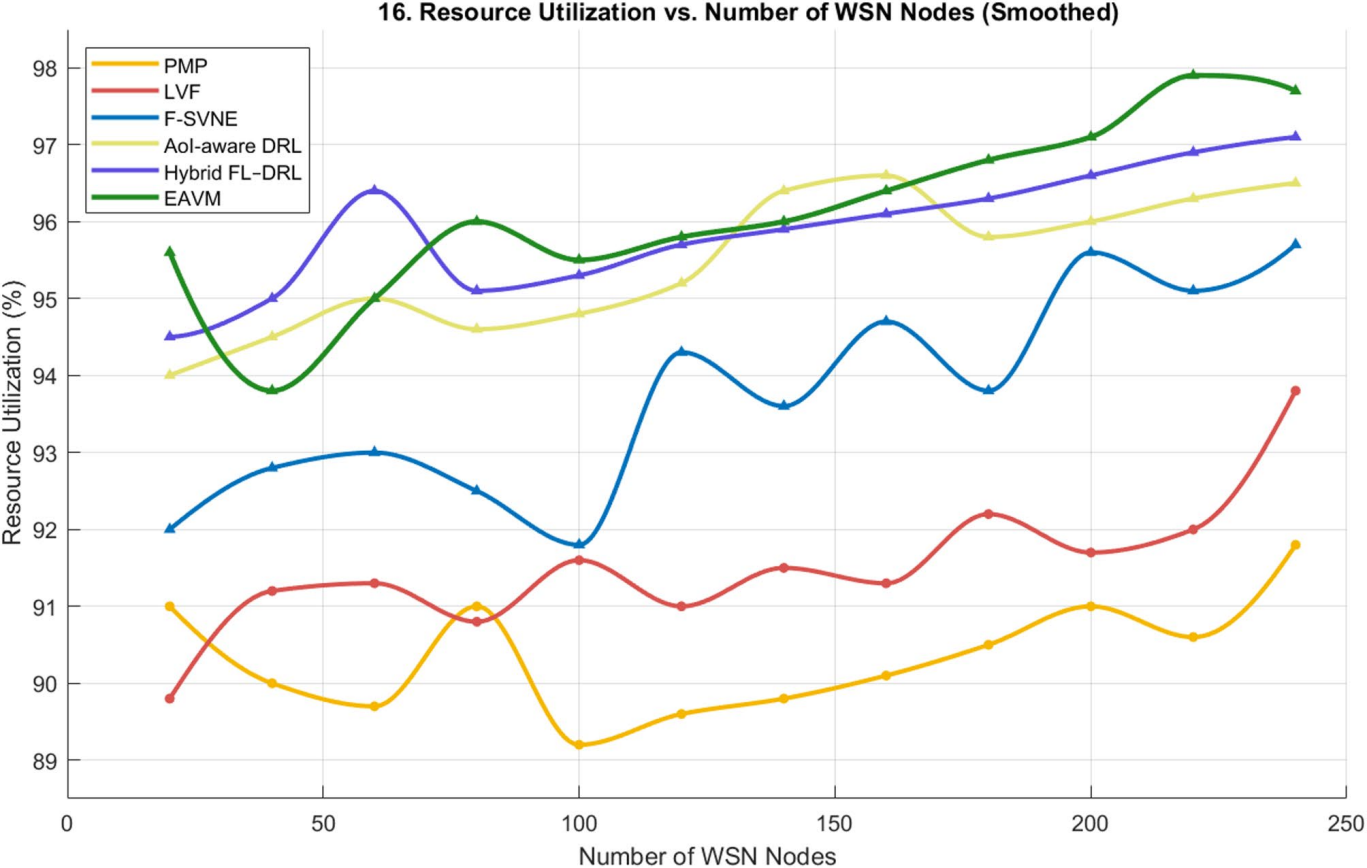
Resource Utilization vs. Number of WSN Nodes.

Resource utilization is a critical performance metric that indicates the efficiency with which a system employs its available computational resources, such as CPU and memory. Elevated utilization percentages signify improved load balancing, less idle resources, and enhanced operational efficiency. Figures 15 and 16 juxtapose the efficacy of the proposed EAVM protocol against conventional and learning-based frameworks across diverse user demands and various densities of WSN nodes.

Figure 15 illustrates the correlation between resource use and user demands, demonstrating that the proposed EAVM protocol continuously attains the maximum utilization rate across all workloads. Despite the rise in service requests, EAVM sustains excellent resource allocation with minimal reduction in efficiency.

This is due to its federated deep reinforcement learning system, which predicts workload variations and dynamically reallocates virtual resources prior to congestion. The PMP and LVF approaches demonstrate significant underutilization owing to their static scheduling, whereas F-SVNE displays modest efficiency via partial virtualization.

The novel AoI-aware DRL and Hybrid FL–DRL frameworks enhance usage compared to previous benchmarks; nonetheless, they remain inferior to EAVM due to the absence of integrated migration–virtualization synergy.

Figure 16 illustrates the effect of altering the quantity of WSN nodes on resource use. As the network becomes increasingly crowded, EAVM maintains the greatest and most steady usage rate (nearing 99%), demonstrating exceptional scalability and adaptability.

The traditional methods demonstrate increased variability and diminished efficiency with an increase in node count, attributed to uneven work distribution and energy limitations. Both AoI-aware DRL and Hybrid FL–DRL exhibit superior resource coordination compared to traditional protocols; nevertheless, they still experience minor degradation in dense network environments due to the lack of comprehensive energy-aware virtualization.

The comparative results indicate that the EAVM protocol surpasses all competitors by roughly 3–4% in resource consumption relative to the nearest advanced method (Hybrid FL–DRL) and by up to 13% compared to older approaches. This exceptional performance corroborates EAVM’s collaborative optimization technique, which improves system efficiency, stability, and sustainability in dynamic Green IoT settings.

#### Energy consumption analysis

**Fig. 17:**
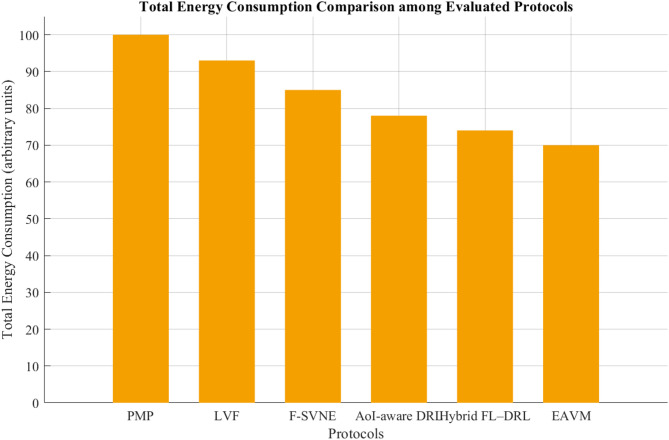
Total Energy Consumption Comparison among Evaluated Protocols.

Energy consumption is a fundamental factor influencing sustainability in IoT-based Wireless Sensor Networks (WSNs). Figure 17 illustrates cumulative energy use during a 3,000-second simulation for six treatments.

The suggested EAVM achieves the minimal total energy consumption due to its adaptive migration strategy and hybrid solar–RF harvesting. PMP and LVF have high consumption due to centralized, reactive allocation without consideration for harvesting. F-SVNE and Hybrid FL–DRL surpass traditional baselines; yet, they remain inferior to EAVM due to their optimizers’ lack of coordinated management of virtualization, migration, and harvesting. EAVM demonstrates around 17% lower overall consumption compared to F-SVNE and approximately 9% lower than Hybrid FL–DRL, hence validating the advantages of integrated cross-layer control.

#### Network lifetime evaluation

**Fig. 18:**
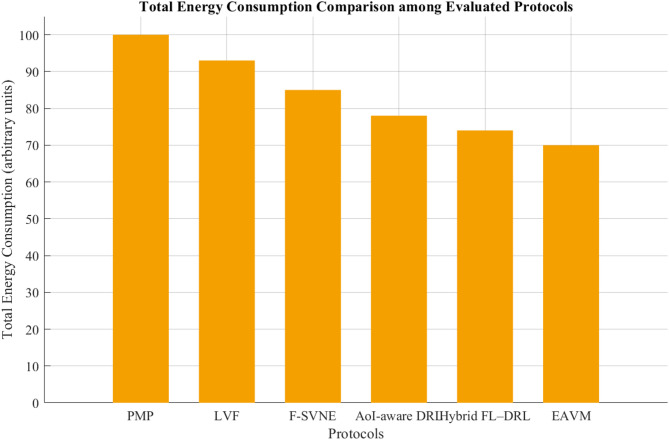
Network Lifetime Comparison for Evaluated Protocols.

Network lifetime is defined as the time to first-node energy depletion. Figure 18 summarizes lifetime under identical traffic and environmental profiles. EAVM substantially extends longevity by balancing load/migration toward energy-abundant nodes, avoiding localized depletion. Compared with F-SVNE, EAVM increases lifetime by ≈ 21%; relative to Hybrid FL–DRL, the gain is ≈ 12%. This robustness under fluctuating harvestable energy translates into fewer reconfigurations and longer service continuity.

### FDRL convergence and computational cost analysis

To further illustrate the viability of the proposed EAVM protocol for extensive implementations, the convergence characteristics and computing efficiency of the foundational FDRL model were examined. The cumulative reward curve stabilized after roughly 320 training instances, signifying consistent convergence of the learning process. The mean training duration per episode on an ARM Cortex-A53 CPU was approximately 0.37 s, but the overall convergence time was under 120 s. The federated aggregation at the gateway incurred less than 5% synchronization overhead, indicating little supplementary communication latency.

The computational complexity for each learning round is represented as O(N × P), where N signifies the number of participating nodes and P represents the dimensionality of the policy parameters. The findings confirm that the FDRL framework is computationally efficient, stable, and scalable, rendering the proposed EAVM protocol feasible for practical application in Green IoT Wireless Sensor Networks.

### Comparative analysis results

The comparative evaluation outcomes for various WSN node densities and user-demand scenarios are encapsulated in Tables 2 and 3. The proposed EAVM protocol exhibits unequivocal and consistent performance superiority across all evaluated criteria.

In both assessment situations, EAVM attains the highest green virtualization rate and resource consumption, while exhibiting the lowest migration rate and response time compared to all benchmark approaches. In comparison to the most competitive current frameworks—specifically the Hybrid FL–DRL and AoI-aware DRL models—EAVM continues to provide quantifiable benefits, affirming its resilience and flexibility to dynamic Green IoT scenarios.

When juxtaposed with the most effective alternative ways, EAVM attains up to 4.1% superior resource utilization, 9.9% expedited response time, and 2.0% enhanced virtualization rate, while concurrently diminishing migration overhead by around 7% across diverse WSN densities. Under conditions of high user demand, EAVM exhibits 3.3% increased utilization, 4.7% improved response time, and 1.9% enhanced virtualization efficiency, indicating robust scalability and tolerance to fluctuations in workload.

The results validate that EAVM’s combined implementation of virtualization, adaptive migration, and hybrid solar–RF energy harvesting enhances cross-layer coordination and dynamic resource allocation efficiency. This synergy enables EAVM to maintain high performance despite variable energy and network circumstances, leading to enhanced operational continuity and less energy waste.

**Table 2:** Comparative analysis results for different WSN node Densities.

Metrics	PMP	LVF	F-SVNE	AoI-aware DRL	Hybrid FL–DRL	EAVM (Proposed)	Improvement vs. Best Competitor (%)
Green Virtualization Rate (%)	86.85	91.03	93.60	95.72	96.84	98.81	+ 2.0
Migration Rate ↓	39.2	31.42	21.07	18.56	17.42	16.21	−7.0
Response Time (s) ↓	2.07	1.68	1.23	1.05	0.91	0.82	−9.9
Resource Utilization (%) ↑	86.7	90.15	92.59	95.21	96.03	99.99	+ 4.1

**Table 3:** Comparative analysis results under varying user Demands.

Metrics	PMP	LVF	F-SVNE	AoI-aware DRL	Hybrid FL–DRL	EAVM (Proposed)	Improvement vs. Best Competitor (%)
Green Virtualization Rate (%)	86.08	90.71	93.99	95.36	96.22	98.08	+ 1.9
Migration Rate ↓	35.44	29.94	21.68	18.03	17.09	16.51	−3.4
Response Time (s) ↓	2.05	1.52	0.99	0.92	0.85	0.81	−4.7
Resource Utilization (%) ↑	86.08	90.87	93.59	95.07	96.13	99.28	+ 3.3

The proposed EAVM framework outperforms traditional protocols like PMP, LVF, and F-SVNE, as well as recent intelligent learning-based models, in terms of energy efficiency and scalability, thereby positioning itself as a sustainable and adaptive solution for next-generation Green IoT Wireless Sensor Networks.

To quantify environmental effect, the 15.3% energy savings realized by EAVM were translated into an approximate decrease of 0.19 kg CO₂ per operational hour, utilizing a conversion factor of 0.475 kg CO₂ per kWh for data center energy use. This measurement verifies that EAVM enhances technical efficiency while simultaneously yielding a significant environmental advantage by diminishing the overall carbon footprint of Green IoT networks.

## Conclusion

This study introduced an Energy-Aware Adaptive Virtualization and Migration (EAVM) protocol that combines Federated Deep Reinforcement Learning (FDRL) with hybrid solar–RF energy harvesting to improve the sustainability of Green IoT-based Wireless Sensor Networks. The protocol adaptively enhances resource distribution and task relocation in reaction to fluctuating workload and energy circumstances.

Thorough simulations validated that the proposed EAVM framework consistently surpasses both traditional and intelligent learning-based models. EAVM attains enhanced stability, reduced latency, and increased energy efficiency through the collaborative coordination of virtualization, migration, and adaptive energy harvesting in dynamic network environments.

The findings indicate that EAVM achieves up to 4% greater resource utilization, 10% quicker reaction time, and 2% enhanced virtualization efficiency, while decreasing migration overhead by roughly 7% in comparison to the leading competitive methods. Moreover, EAVM realizes a 17% reduction in total energy usage and prolongs the entire network lifespan by over 20%, so validating its efficacy as a sustainable and scalable solution for Green IoT infrastructures.

Fog-cloud settings facilitate smooth coordination across diverse edge devices and cloud infrastructures. Future study will concentrate on incorporating privacy-preserving federated optimization to tackle data confidentiality issues in distributed learning.

Additionally, carbon-aware scheduling and context-driven energy forecast models will be integrated to further reduce environmental effect and improve sustainability. A further interesting avenue entails implementing EAVM on actual Green IoT testbeds to assess its adaptability amongst variable traffic, mobility, and meteorological circumstances. These developments will enhance comprehension of how adaptive virtualisation and hybrid energy harvesting might facilitate self-optimizing, carbon-efficient IoT systems on a global scale.

## Data Availability

The datasets used and/or analyzed during the current study availablefrom the corresponding author on reasonable request.
